# The development of physical characteristics in adolescent team sport athletes: A systematic review

**DOI:** 10.1371/journal.pone.0296181

**Published:** 2023-12-21

**Authors:** Lars M. Tingelstad, Truls Raastad, Kevin Till, Live S. Luteberget

**Affiliations:** 1 Department of Physical Performance, Norwegian School of Sport Science, Oslo, Norway; 2 Carnegie School of Sport, Leeds Beckett University, Leeds, England; University of Montenegro, MONTENEGRO

## Abstract

**Background:**

Physical development during adolescence is crucial for athletes in team sports, as it prepares them for the high sport demands at the senior level. While physical development in non-athletes are well-documented, a comprehensive understanding of adolescent athletes’ development, including the potential effects of team sports participation and training load, is lacking.

**Objectives:**

The study aimed to investigate the development of physical characteristics in team sport athletes during adolescence (12–20 years) and explore the impact of training load.

**Methods:**

A systematic search of the databases PubMed, SPORTDiscus and Web of Science were conducted combining keywords related to physical characteristics, youth athletes, team sport and study design. Criteria for inclusion were: (1) team sport athletes aged 12–20 years, (2) cross-sectional or longitudinal designs investigating physical characteristics, (3) comparisons across different age groups, (4) peer-reviewed original article, (5) written in English, and (6) available results from physical testing. Results were normalized and weighted based on sample size.

**Results:**

176 eligible articles were identified. The results showed consistent annual improvement in most physical characteristics from 12 to 16 years for both sexes (e.g., boys: lower body strength 14.3%; intermittent endurance 11%; countermovement jump height 6.7%; change of direction 2.8%; 30 m sprint 3.6%, and girls: lower body strength 9.4%; intermittent endurance 12.1%; countermovement jump 4.7%; change of direction 3.3%; 30 m sprint 1.9%). Only 4 studies investigated the effect of training load on physical development.

**Conclusions:**

Although both sexes consistently improved through adolescence, girls tended to have less pronounced physical development compared to boys, likely due to lower increase in lean mass and limb length. The existing evidence do not definitively establish whether team sports participation, compared to studies examining non-athletes, or training load have an additive effect on physical development during this period.

## Introduction

Team sports are typically characterized by frequent bouts of efforts ranging from low to maximal intensity [1–4]. Consequently, athletes must be able to generate high amounts of force and power to jump, sprint, accelerate, change direction, and perform explosive actions like kicking and throwing [5–7]. Research studies consistently show elite players to outperform sub-elite peers in several physical parameters [8–12]. Furthermore, the ever-increasing physical demands of sport emphasize the importance for athletes to possess well-developed physical characteristics to achieve high-level performance at the senior level [13, 14]. Physical characteristics are therefore highly valued and extensively used as selection criteria in talent programs within team sports [15].

To support adolescent athletes in their long-term development process, knowledge regarding typical development trends for physical characteristics during the adolescent years is crucial. This knowledge would contribute to the monitoring and evaluation of adolescent athletes’ progress, facilitating the identification of their strengths and weaknesses, optimizing the design of effective training programs, and evaluating training interventions [16]. Understanding the various factors influencing the development process can enhance the customization of training programs to optimize the development of adolescent athletes. Growth and maturation are the main drivers for the development of physical characteristics during this period [17], with increases in body mass and height, fiber-type differentiation, resting adenosine triphosphate and creatine phosphate levels, increased androgen concentrations, and architectural development of musculotendon units [18], all contributing to the development of different physical characteristics. However, the specific effects of aspects related to training load remain uncertain in this population. Adaptations to training have been extensively studied in adults, both in the context of the development of physical characteristics and injury prevention [19, 20]. However, less attention has been given to understanding the unique responses of young athletes to exercise stimuli [21], highlighting the need for further exploration in this area.

Despite the extensive research conducted on physical characteristics in boys during adolescence [9, 22–27], there is currently no systematic review that comprehensively summarizes the literature on team sport athletes, specifically encompassing both boys and girls. This knowledge gap is significant, considering that studies conducted on non-athletes have consistently demonstrated notable differences in the development between the sexes during the early to late stages of adolescence [17, 28–30]. Research findings on non-athletes indicate that boys tend to demonstrate greater improvements in physical characteristics during adolescence, while girls often reach a plateau shortly after puberty; typically around the age of 13–15 years [17, 28–31]. These discrepancies may be attributed, among other things, to the longer and more potent maturity processes impacting physical development in boys [17]. However, it remains uncertain whether these patterns hold for athletes involved in team sports. If team sport athletes follow similar development trajectories, their natural progression may slow down during late adolescence. This could highlight a need for more targeted physical training programs to adequately prepare them for the demands of the senior level. Therefore, the primary aim of this systematic review was to investigate the development of physical characteristics during adolescence in team sport athletes in both boys and girls. Additionally, the effects of training load on the development of physical characteristics were investigated.

## Methods

This systematic review was conducted following the Preferred Reporting Items of Systematic Reviews and Meta-analyses (PRISMA) statement [32] and registered on Open Science Framework (Registration https://doi.org/10.17605/OSF.IO/9A86G). Completed PRISMA-P checklist can be found in S1 Checklist. The aim was to evaluate the scientific literature investigating the development of physical characteristics among adolescent team sport athletes and investigate the impact of training load on the development of physical characteristics. A search of databases (SPORTDiscus, PubMed, and Web of Science) for eligible published articles was performed on December 7, 2021, combining keywords related to physical characteristics, adolescent athletes, team sport, and study design (Table 1). A second systematic search was performed on January 26, 2023, to include any new articles published within the previous year since the first search. No restriction on the year of publication was applied, according to PRISMA recommendations [33]. All cross-sectional, longitudinal, and mixed-longitudinal studies were included. Studies were categorized as mixed-longitudinal if they involved tracking changes over time in a sample of participants, but unlike longitudinal studies, not all participants were the same at every measurement point.

**Table 1:** Search terms used to find eligible articles.

	Search term	
	**Physical characteristics**	(physical OR “physiological testing” OR “performance tests” OR aerobic* OR “endurance” OR strength OR “soccer physiology” OR “exercise test*” OR anthropometric* OR “body composition”)
AND	**Age**	(youth OR adolescent OR adolescence OR child* OR academy OR “young adult” OR “adolescent development”)
AND	**Team sport**	(“team sport” OR handball OR soccer OR football OR team handball OR “field sport*” OR “court-based sport*” OR rugby OR hockey OR cricket OR basketball OR “field conditions” OR “netball” OR technical tactical skill)
AND	**Study design**	(longitudinal OR “long term” OR “cross-sectional” OR “quasi-experimental” OR “prospective observational” OR “mixed longitudinal”)

AND is used between each search term to include all the variables

### Study selection

**Fig 1:**
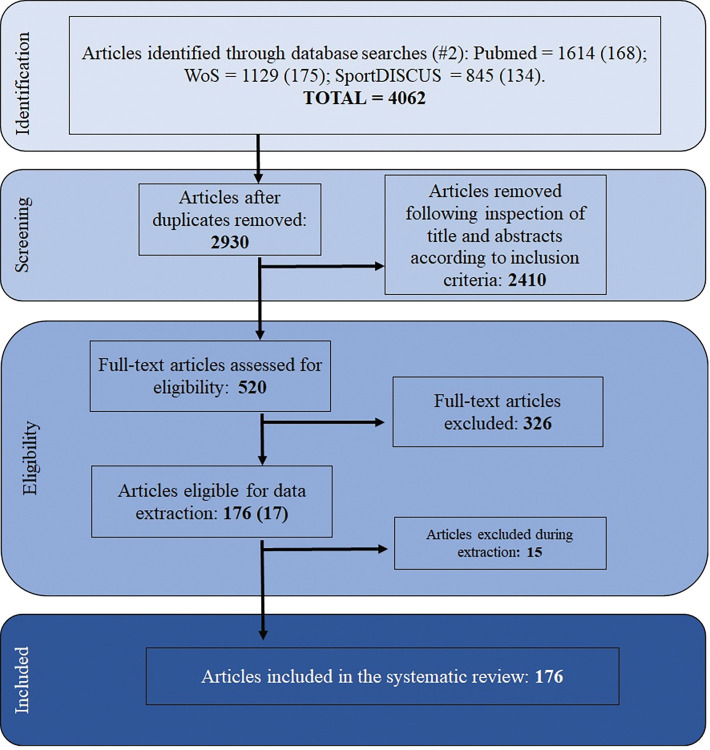
PRISMA flow chart. 14 studies were added after updated search.

Following the initial search, the results were exported to EndNote library (Endnote X9, Clarivate Analytics, USA), and duplicate articles were removed. The remaining articles were then uploaded to DistillerSR (https://www.distillersr.com/, Ottawa, Canada) and two independent reviewers (LSL, LMT) independently screened the content of the titles and abstracts against the predefined inclusion-exclusion criteria (Table 2). Both cross-sectional and longitudinal study designs were included to allow a larger dataset to better investigate the developmental trajectories of team sport athletes during adolescence. The age range of 12 to 20 years was chosen as 12 years of age is when official competition and differentiation between levels begins in Norway. Full texts of the included articles were later retrieved and reviewed. Any discrepancies between the two researchers at this stage were discussed, and if an agreement could not be reached, it was resolved by a third reviewer (TR). An overview of the articles included and excluded in each stage of the screening process is shown in the flow chart (Fig 1).

**Table 2:** Inclusion criteria for articles eligible for the review.

Criteria	Inclusion
1	Studies with a sample of adolescent team sport athletes (between 12 and 20 years old)
2	The design was either cross-sectional with different age groups or a longitudinal design lasting at least one year investigating speed, change of direction ability, strength, power, and/or endurance capabilities
3	Comparison between different age groups (excluding groups comprised by different ages)
4	Peer-reviewed original article
5	Full text in English available for screening
6	Results from physical testing were available

### Methodological quality assessment

The included articles were assessed independently by the two main reviewers using the “quality assessment tool for observational cohort and cross-sectional studies” developed by the National Heart, Lung and Blood Institute (NHLB, 2021). Some questions were modified, and some were not included due to being irrelevant (see S2 Table for the included questions). Discrepancies or conflicts were either resolved through discussion or the involvement of a third reviewer if an agreement was not met. Results are presented in S3 Table. As both cross-sectional and longitudinal studies were included, it’s worth noting that certain questions (7 and 13) were only relevant to the longitudinal studies. This distinction led to a scoring potential of up to seven points for the cross-sectional studies and up to nine points for the longitudinal studies.

### Data extraction

Data were extracted using a specifically designed standardized Excel spreadsheet, which included publication data (authors, year of publication, sample information, duration, methodology, sport, level of competition, sex, country), anthropometrics (height, weight, body composition), physical characteristics tests with output measures for each age group (sprint, change of direction (CoD) ability, endurance, lower and upper body strength, vertical jump), training load (hours of training) and maturation data. WebPlotDigitiser (Version 4.1, Pacifica, California, USA) [34] was used to extract means and measures of variance (standard deviation) from figures when tables were not present. To avoid intervention bias, only the baseline score was extracted from studies that included interventions. The extraction was carried out by both reviewers, and the findings were merged into a single document and later examined for conflicts. Conflicts not resolved between the two researchers were discussed and resolved by a third reviewer if an agreement was not reached. After extraction, 21 papers were discovered to not report the sex of their sample. The authors were later contacted to retrieve this information.¨Extracted data from all included papers can be found in S1 Data.

### Strategy for data synthesis

All data were analyzed using descriptive statistics and reported as annual differences between age groups, (i.e., either average for age range [12–16 years] or between two consecutive age groups [e.g., 13–14 years]). The following tests for each physical characteristic were chosen for analysis due to being the most utilized tests:

- Sprint: 10 and 30 m.
- Vertical jump: CMJ.
- Intermittent endurance; YYIR1 and 20-m multistage test.
- CoD ability: Agility 505, 10 x 5 m shuttle run, and 5 x 10 m shuttle run.
- Upper body strength: handgrip.
- Lower body strength: a large diversity in lower-body strength tests in the included studies rendered it necessary to include several different tests (Table 3).

**Table 3:** The different variations of strength tests included in the analysis and the number of studies using each test.

Test	n
Isokinetic PT knee extension 60°/s	8
1 RM back squat	7
Isometric mid-thigh pull peak force	4
Isokinetic PT knee extension 180°/s	3
Isokinetic PT knee extension 240°/s	3
Knee extension ISO	3
1RM front squat	3
Iso knee extension 60°/s	1
Isokinetic PT knee extension 225°/s	1
Isokinetic PT knee extension 240°/s eccentric	1
Isokinetic PT knee extension 60°/s eccentric	1
Isokinetic PT knee extension 90°/s	1
Leg extension	1
5RM squat	1

The results from each of these tests were then merged through normalization (described in detail below). Figures were made in GraphPad Prism (Version 9.2.0, GraphPad Software Inc, San Diego, CA, USA). Tables and all calculations for yearly changes were analyzed in Microsoft Excel.

#### Normalization and weighting of data

To accommodate the diverse range of tests and methods utilized in the studies, a normalization process was implemented to standardize the data to a common scale. This approach allowed for the inclusion of a larger volume of data, promoting a more comprehensive analysis. The normalization process involved dividing the test results for each age group with the result from a reference age group. The reference age group was selected for each test individually and was set to the most frequently included age group for each physical characteristic. Studies that did not include this group, were normalized to the closest age group (to the reference age). Each result was then weighted relative to the group’s sample size.

## Results

### Study characteristics

**Fig 2:**
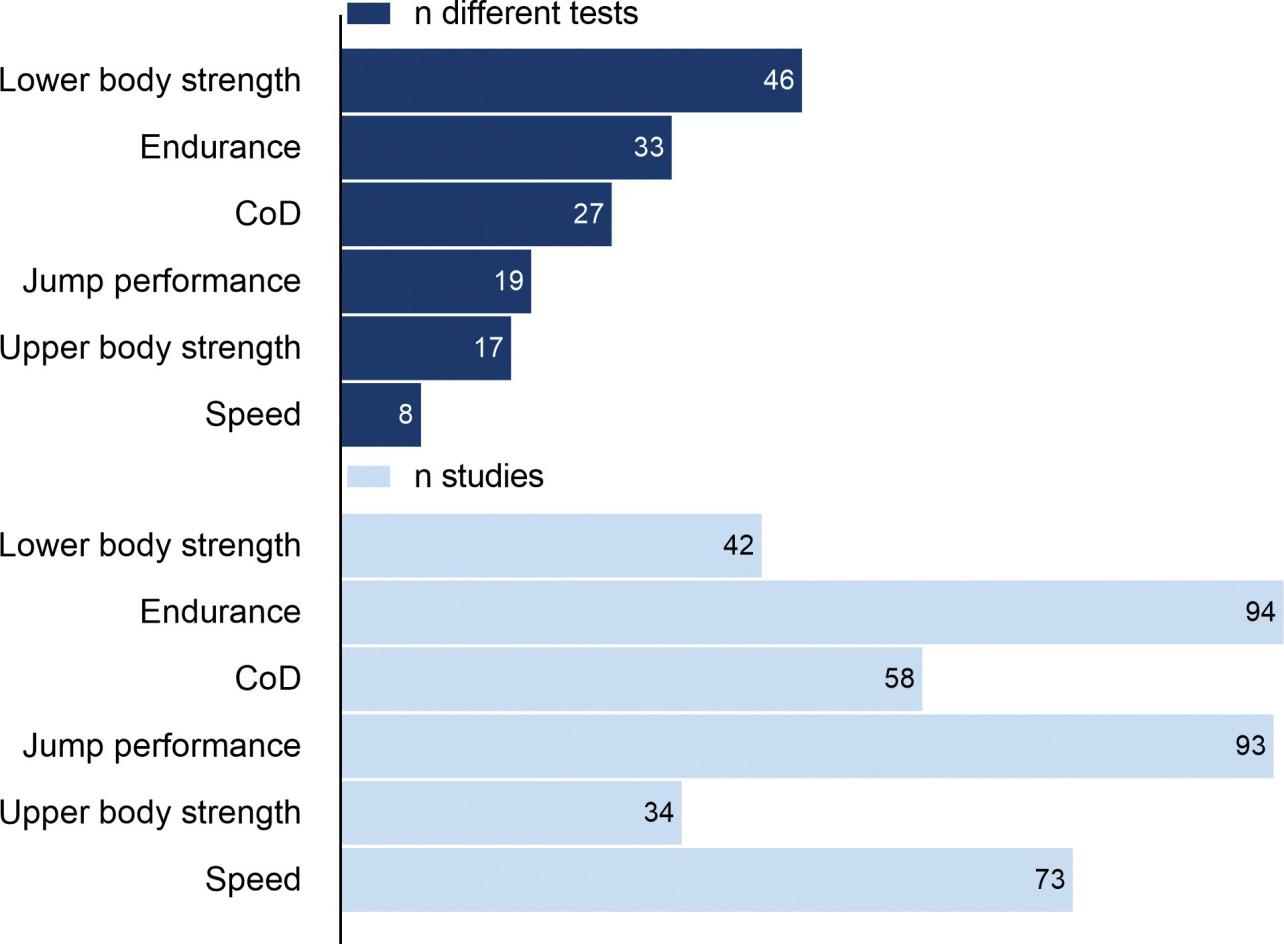
Variety in testing methodologies: Number of different tests for each physical characteristic and the number of studies each characteristic was measured. CoD: change of direction.

Across the 176 articles included in the study, 85% (n = 151) had a sample consisting of only boys, 8% (n = 14) had a sample consisting of only girls, and 7% (n = 12) included both sexes. The sample sizes ranged from 9–13,869 boys with a median of 130 participants and 22–1,832 girls with a median of 93. The total for each sex was 56,665 boys and 4,616 girls. More than half (55%, n = 104) had a cross-sectional study design, 22% (n = 39) were mixed-longitudinal and 19% (n = 34) were longitudinal. There were 11 different sports represented, and the most common are presented in S1 Fig. A large majority of the studies were conducted in Europe (n = 123), and England (n = 24), Australia (n = 17), Belgium (n = 13), Brazil (n = 13), Portugal (n = 13), and Germany (n = 12), were the most common countries. Seventeen (9%) studies included measures of training load, and 54 studies (31%) provided information about maturation status, reported in one of the following ways: years from peak height velocity (YPHV) (n = 24), age at peak height velocity (APHV) (n = 12), skeletal age (n = 9), sexual maturation (n = 5), predicted adult height (n = 2), biological age (n = 1), and maturity ratio (n = 1). This variation in methodology, additionally to limited studies including different maturity groups within the same age cohort, made it impossible to summarize the literature to address differences in development between different maturity groups. The number of different tests used for each physical characteristics and the number of studies including each is summarized in Fig 2. A summary including author, country, study design, duration, sample size, age groups, competitive level, sport, and tests used for all studies are reported in S3 Table. The number of studies included for each age group and physical characteristic is available in S4 Table.

### Methodological quality

The methodological quality assessment scores are presented S2 Table, averaging a score of 70 ± 13%, ranging from 43% to 100% for the items evaluated. The majority of all the studies addressed questions 1, 2, 4, 7, 9, and 11 (n = 69–100%). However, participation rate of eligible subjects was not possible to determine in most studies (1%), few studies included a sample size justification (3%), and loss to follow-up after baseline was, when applicable (11%), usually not reported or could not be determined.

### Physical characteristics across age-groups in team sport athletes

**Fig 3:**
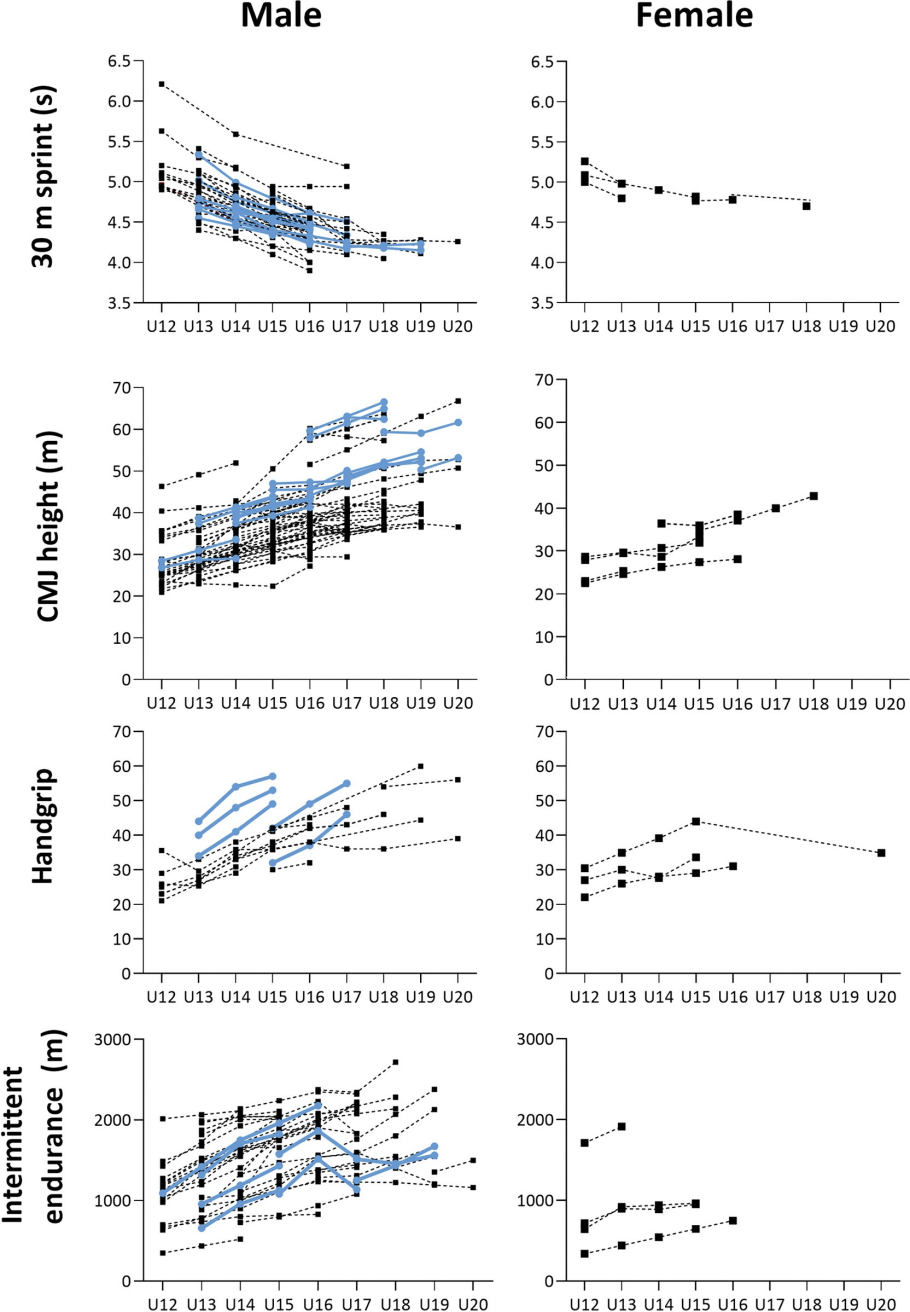
Yearly performance measured in all studies included for 10 m sprint, CMJ, intermittent endurance and handgrip strength. Boys are the left figures, and girls the rigth. Blue lines represent longitudinal studies, and black dotted lines are the cross-sectional studies. CMJ: countermovement jump height.

Fig 3 provides an overview of all individual studies investigating 30-m sprint, CMJ, handgrip strength, and intermittent endurance. It visually presents the variations and trends in the development of physical characteristics observed among different age groups in these studies. Notably, the figure also illustrates that there are no substantial differences in the results between cross-sectional and longitudinal study designs overall, though longitudinal studies seem to have less variation. No longitudinal studies were found examining changes in physical characteristics in girls for the selected tests.

#### Anthropometrics (height, body mass, body fat)

**Fig 4:**
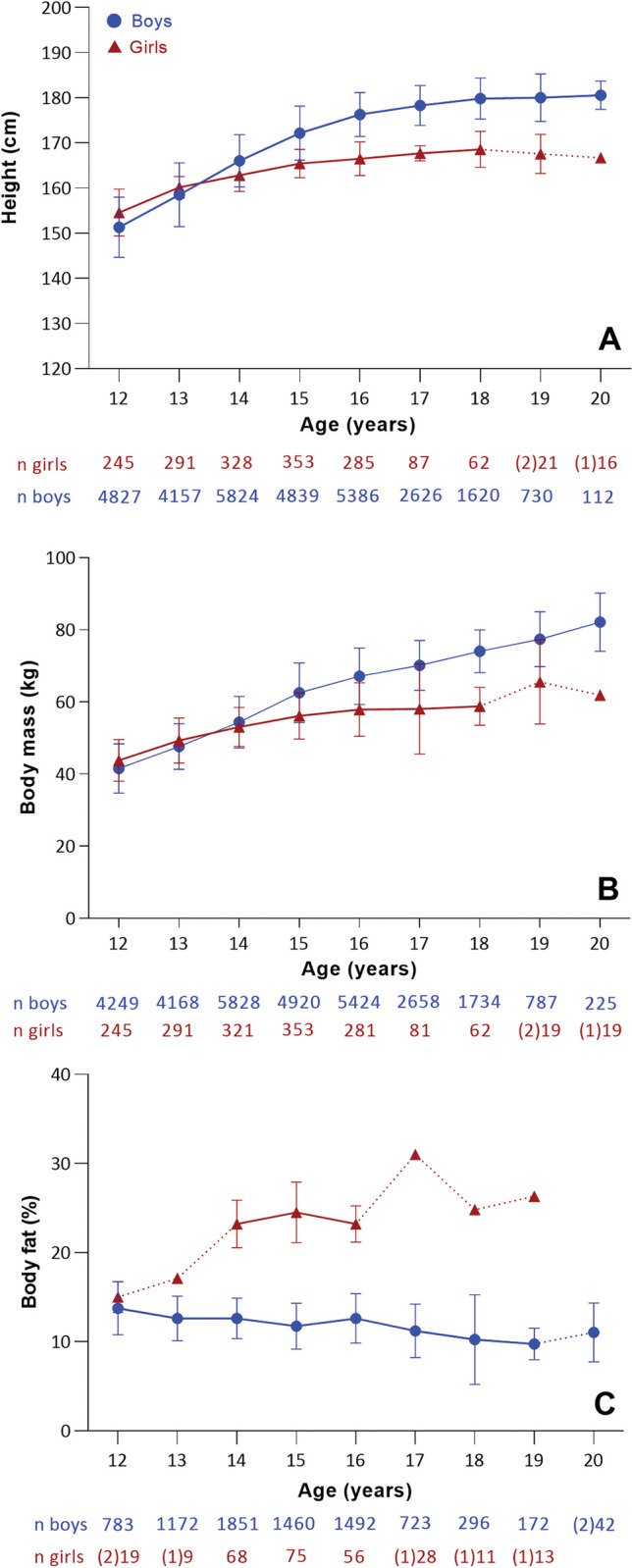
Weighted data for height (A), body mass (B) and percentage body fat (C). The sample size for each age group is indicated below the x-axis. The numbers in parentheses represent the available studies for the respective age group. Dotted lines highlight age groups with two or fewer studies.

The results from the analysis of anthropometric characteristics, show boys to have a notably greater average yearly increase in height from 12–20 years (2.3%) compared to girls (1%) (Fig 4A). Moreover, both boys (0.6%) and girls (0%) show minimal growth in height from 16 to 20 years. Changes in body mass follows the same trend, with boys demonstrating greater yearly increase from 12 to 20 years (9%), compared to girls (4.6%) (Fig 4B). Most growth occur during adolescence and seems to plateau after the age of 15 years. In terms of percentage body fat, boys demonstrate an average yearly reduction of 2.4% (0.34 percentage points) from 12 to 20 years, while girls have a yearly increase of 10% (1.63 percentage points) (Fig 4C).

#### Lower body strength

**Fig 5:**
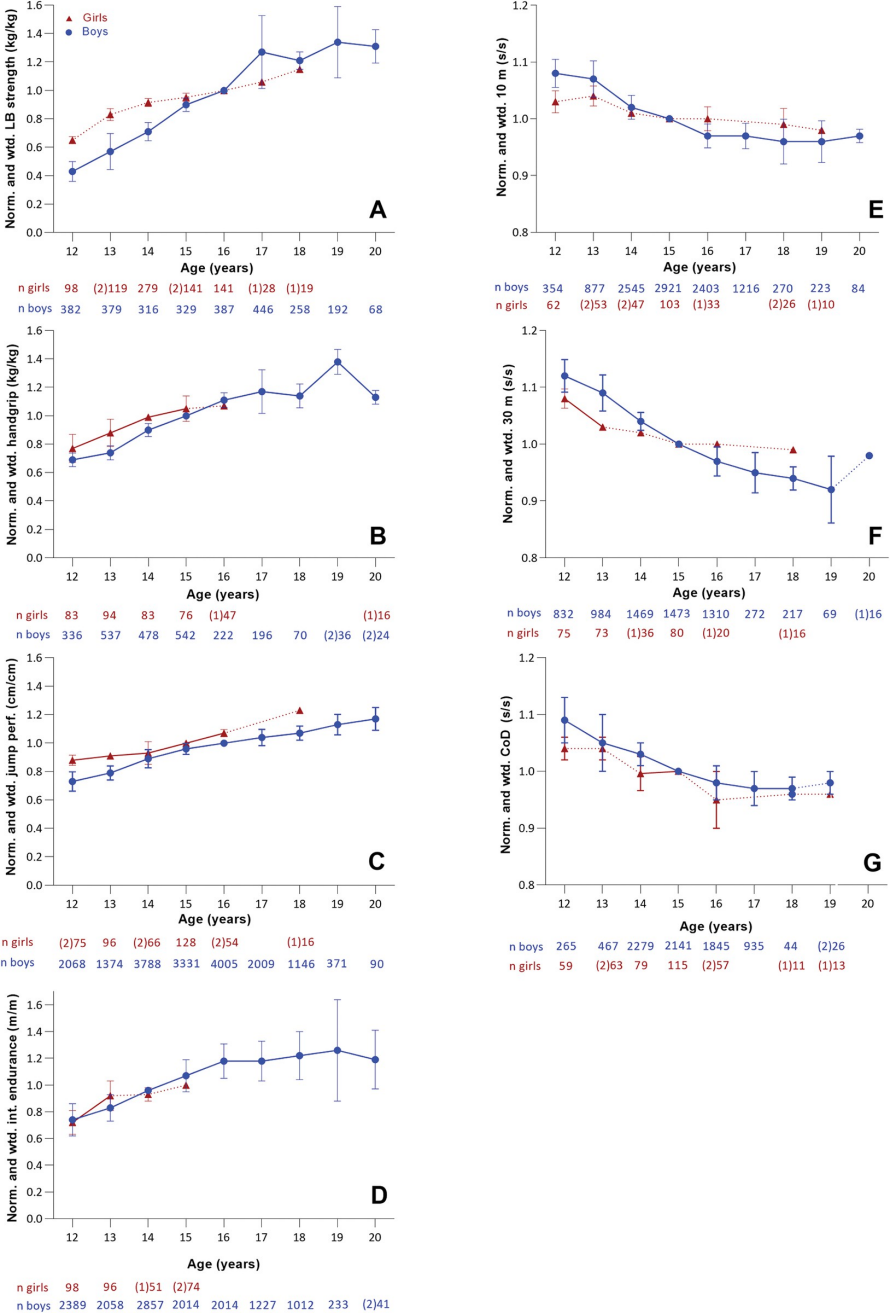
Weighted data for lower (A) and upper (B) body strength, CMJ (C), intermittent endurance (D), 10-m sprint (E), 30-m sprint (F), and CoD (G) results. The sample size for each age group is indicated below the x-axis The numbers in parentheses represent the available studies for the respective age group. Dotted lines highlight age groups with low samples. Norm: normalized. Wtd; weighted. LB: lower body. CoD: change of direction. Int: intermittent. Perf: performance.

The following (normalized and weighted) results for physical characteristics are presented solely in terms of relative development, and do not describe absolute performance. A consistent pattern of improvement in lower body strength can be observed during the adolescent years, with the greatest improvement occurring from 16 to 17 years (27.1%) (Fig 5A). From 12 to 17 years, there is a relatively steady phase of yearly improvement (16.8%), followed by a plateau to 20 years. Girls generally exhibited lower average yearly changes in lower body strength compared to boys. From 12 to 17 years, girls demonstrated a yearly improvement of 8.2%, with the greatest rate occurring from 12 to 13 years at 18%.

**Upper body strength.** Among boys, a consistent yearly improvement of 9.9% was observed from 12 to 16 years, with the greatest rate of improvement occurring from 13 to 14 years (15.9%) (Fig 5C). Following this period, there was a gradual decline in the yearly rate of improvement until 18 years. From 18 to 20 years, data from a limited number of studies suggests a large increase (23.5%) followed by a substantial decrease (24.7%). The average yearly improvement between 12 and 16 years was lower in girls compared to boys, with 7.3%. The greatest improvement was observed from 12 to 13 years (11.1%), followed by a gradually lower rate until 16 years.

#### Linear sprint performance (10 and 30 m)

The downward trend in Fi. 5 (B and D), presenting linear sprint performance, corresponded to improved sprint times (e.g., a reduction in the time used to complete the distance). An average yearly improvement of 1.4% and 2.8% was observed in boys for the 10 m (Fig 5B) and 30 m sprint (Fig 5D) respectively between the ages of 12 and 19 years. This was greater than the improvement in the 10 m sprint observed in girls of 0.8%. Both sexes demonstrated their greatest rate of improvement between 12 and 16 years with an average of 2.6% and 3.6%, and 0.9% and 1.9% respectively for boys and girls in 10 m and 30 m sprint.

#### Change of direction ability

The average yearly improvement in CoD for the boys between 12 and 16 years (2.8%) and 12 and 19 years (1.6%) was comparable to that observed in the 10 m sprint (2.6% & 1.4%) (Fig 5F). The most significant yearly improvement was observed from 12 to 13 years, at 4.1%. From 18–19 years a decline in performance of 1.1% was observed. Girls demonstrated a slightly lower average yearly improvement from 12 to 16 years (2.3%), though with greater variation. No improvement was observed from 12 to 13 years and 14 to 15 years, while from 13 to 14 years and 15 to 16 years, yearly improvements of 4.9% and 5.3%, respectively, were observed. The one study investigating girls aged 18 to 19 years reported a 0.2% improvement.

#### Jump performance

Boys demonstrated large improvements during their early adolescent years with a 9.3% increase from 13 to 14 years, and an average yearly improvement of 6.7% from 12 to 16 years (Fig 5E). The yearly improvement gradually decreased until 18 years, but a slight increase is observed for 19s and 20s. In contrast, girls demonstrated their greatest rate of yearly improvement from 14 to 15 years (7.2%), and an overall yearly improvement of 4.7% from 12 to 16 years.

#### Intermittent endurance

Boys demonstrate an overall yearly improvement of 5.8% from 12 to 20 years. From 12 to 16 years, boys demonstrate an average yearly improvement of 11%, with the greatest rate occurring from 13 to 14 years (13.1%) (Fig 5G), Following this period of rapid development, there is a diminishing rate towards late adolescent (2.8% 16–19 years). A reduction in performance of 6.2% was observed from 19 to 20 years in boys, however this was only based on two studies. Intermittent endurance in girls was only investigated in a few studies (n = 4), but results demonstrate a yearly improvement of 11% from 12 to 15 years, similar to that of boys (9%). The greatest yearly increase in performance was observed from 12 to 13 years (21%).

### Training load

Of the 176 articles included, 17 (9%) measured training volume, either as yearly hours (n = 4), weekly hours (n = 12), or both (n = 1). Furthermore, four studies [35–38] specifically investigated the impact of training on physical development; all reporting a significant effect of training. Baxter-Jones et al. [35] did not find any effect of additional hours of training per se, but noted that improvements in maximal oxygen uptake (VO_2_-max) may vary depending on the type of sport. The remaining studies found that an increase in either weekly [36] or yearly [37, 38] training hours improved performance in endurance. All the studies used a multilevel model analysis to assess the effects of training volume on physical performance. No study examined other aspects of training load beyond training volume, and only one study [38] looked at physical characteristics other than endurance (i.e., repeated sprint ability, agility, power).

## Discussion

This study presents the first systematic review investigating the physical development of adolescent boys and girls participating in team sports from 12 to 20 years. Understanding the typical developmental trends of physical characteristics in adolescent team sport athletes is crucial for bridging the transition from adolescent to senior levels. However, there is a gap in the literature regarding longitudinal physical development during adolescence, especially among girls. The findings of this study indicate a consistent annual improvement in physical characteristics from 12 to 16 years, followed by a plateau in the rate of development in late adolescence (i.e., 16 to 20 years). It is important to acknowledge the considerable variability in the rate of change between age groups, particularly in the older age groups where fewer studies were available for analysis. Although girls demonstrated improvement in most physical characteristics, it was for the most part at a lower rate than observed in boys. However, the limited number of studies investigating girls and older age groups limits the strength of these observations. To provide an even more holistic view, it is important to consider the influence of maturation on physical development in adolescents. Nevertheless, maturation was not consistently measured or reported across the included studies, which posed a challenge to conduct a comprehensive analysis on maturation status.

### Physical development in adolescence

#### Anthropometrics

Findings showed that both boys and girls team sport athletes demonstrated increases in height and body mass during adolescence, similar to non-athletes [39], with boys exhibiting greater yearly increases from the age of 12 to 20 years compared to girls. This is likely due to the differential effects of growth and maturation between the sexes, with boys experiencing more pronounced growth during puberty and also benefiting from an approximate two year longer pre-adolescent growth period, resulting in the greater increases in height and body mass [17]. Although both sexes demonstrated increases in body mass, girls have a significant increase in body fat from 12 to 15 years before stabilizing (Fig 4B), whereas boys remain rather stable until 16 years, before a reduction is observed. The overall increase in body mass in boys is due to greater increases in lean mass (skeletal tissue) [17]. Boys also see a further reduction in body fat beyond adolescence, with senior elite rugby players found to have less fat mass and more fat-free mass than junior elite players [40], which may be advantageous for horizontal and vertical acceleration and a reduced metabolic cost of exercise [41]. The observed sex differences in the development of these anthropometrical variables are likely to have implications for the divergent performance outcomes observed between the sexes. This aspect will be further explored and discussed in the subsequent sections.

#### Muscular strength

Existing research on non-athletes indicates that boys and girls have similar levels of strength until boys reach puberty [39]. However, whereas boys continue to develop strength, girls tend to plateau, resulting in increased sex differences as they age [31, 39]. While the findings from this study partially align with these observations, there are some contrasting results. It was observed that girls progressively increased lower body strength during adolescence (Fig 5A), although with a smaller magnitude than boys. During puberty, boys undergo a strength spurt characterized by a rapid increase in circulating hormones, including testosterone and growth hormones. These hormones stimulate protein-synthesis pathways contributing to increased muscle growth, which in turn facilitates increased muscle strength and power production [42–44]. Girls do not have the same increase in circulating testosterone, and consequently exhibit a greater increase in fat mass and a comparatively smaller magnitude of muscle growth and strength development than boys [39], leading to the observed divergence in strength development. This divergence in development has been observed in elite adolescent athletes, particularly in swimming, running, jumping track and field events, where testosterone has been identified as the main factor explaining the performance gap between boys and girls starting around the age of 12–13 years [45].

Differences in strength training practices around this period is another potential factor contributing to the observed differences between sex and age [17, 27, 46]. It has been suggested that girls tend to be less involved in strength training from an early age compared to boys [17, 27, 46], which could influence their rate of strength development [27, 47, 48]. However, an increase in exposure to strength training during late adolescence for girl team sport athletes [49] could explain the continuous improvement in lower body strength until 18 years observed in this study, contradicting previous research on both athletes and non-athletes, which reported a plateau in lower body strength from 15 years of age [31, 39, 50] The inclusion of strength training during earlier stages of development could potentially reduce some of the gap observed between boys and girls and progress lower body strength beyond the previously observed plateau. However, it is important to note that further research is needed to validate these findings and explore the potential underlying mechanisms contributing to the observed differences in strength development, and the effects of earlier implementation.

#### Sprint, CoD, and jump performance

In general, both boys and girls in team sports progressively improved sprint performance, CoD ability, and jump performance during adolescence (Fig 5B and 5D–5F), comparable to that observed for non-athletes [17, 31, 51], and athletes in other sports [45, 52]. The most rapid improvement observed during the early stages of adolescence is largely due to the effects of maturity-related processes occurring during this period. An increase in limb length is associated with increased stride length and frequency [53–56], which are key determinants of sprinting ability [57]. Furthermore, the development of lean mass and muscle strength play a crucial role in sprinting, jumping, and CoD performance [58]. Increased muscle strength enables athletes to generate greater power and force, allowing for more explosive movements [59]. This likely explains the similar development in both 10 m sprint and CoD ability, considering both are linked to relative strength and the ability to accelerate and decelerate quickly, as supported by previous studies [27, 60]. As boys tend to have more pronounced maturity-related changes compared to girls, this explains the divergent sex difference in physical performance and development observed during this period [45, 52]. The slower development observed during the later stages of adolescence is likely associated with the attainment of a more mature state, wherein the physiological changes resulting from maturity-related processes become less prominent [17].

#### Intermittent endurance

Endurance in team sports is the ability to sustain high-intensity efforts and recover intermittently throughout a game and is an important quality for maintaining a high degree of intensity and technical/tactical skills [61]. In line with the general increase in VO_2_-max observed in adolescents [17], intermittent endurance performance in team sport athletes also continuously improves until about the age of 16 years (Fig 5G). This is not surprising as intermittent endurance measured with the YYIR1 and multistage fitness test is correlated with VO_2_-max [62]. As boys’ approach full maturity at around 16 years, their development in VO_2_-max typically stagnates, likely due to the loss of maturity-related effects, such as increase in height and lean mass, which are closely linked to improvements in absolute VO_2_-max [39]. Consequently, additional training is likely necessary to further improve intermittent endurance performance. Improving linear speed and CoD, would also likely improve performance, especially as test speed increase, placing higher demands on these attributes [61].

Interestingly, while non-athlete girls typically demonstrate a decline in VO_2_-max around 14–15 years of age [39], findings in one study [63] suggest that intermittent endurance continues to improve until the age of 16 years, similar to boys. This aligns with the observations made by Tønnessen et al. [2015], who reported improvements for girls in track and field in 800 m performance even up to 18 years of age. This could be due to the difference in tests performed. A recent study by Landgraff et al. [64], demonstrated differences in development between VO_2_-max and endurance performance during adolescence, where an improved performance was observed, but no change in VO_2_-max. Considering the YYIR1 and multistage fitness test analyzed in this this review are performance based (e.g., dependent of multiple physical characteristics) this could help explain the observed difference between development of VO_2_-max in non-athletes and endurance in athletes in this review. This would imply that more factors than VO_2_-max influences performance, such as specific adaptations in the musculature, which could be more susceptible to training.

The lack of difference in improvement between boys and girls is however in contrast to previous studies on non-athletes and athletes in other sports, which consistently show clear divergence in performance occurring at the onset of puberty in boys [45, 52]. Given that girls typically demonstrate a greater increase in fat mass, which is associated with a decline in VO_2_-max, it would be expected to observe less yearly improvement in girls than boys [64–66]. However, it is important to note that there is a significantly fewer studies including girls, and none with a longitudinal design, which may contribute to the unexpected results.

### The impact of training load and team sport participation

The influence of training load on the development of physical characteristics was examined in only four studies [35–38]. These studies reported positive effects with training; however, there is no consensus whether these effects are primarily attributed to training volume [36–38], or the specific training regimen associated with the sport [35]. This aligns with the findings of Wrigley et al. [67], who reported significantly greater improvement in various physical performance measures among a group of academy soccer players (U12 to U16 collectively) compared to non-academy players. On the other hand, Landgraff et al. [64] found no significant difference in longitudinal changes in VO_2_-max between endurance athletes and team sport athletes, although the endurance athletes demonstrated greater improvement in performance, measured as running-time to exhaustion. These contrasting results suggest that factors beyond training volume, such as content, intensity, specificity, and quality [68] may play a more significant role in influencing the outcomes than training volume per se. In a recent systematic review conducted by Dudley et al. [69], the relationship between training load and physical qualities in adolescent athletes was examined. The review revealed moderate evidence supporting a relationship between resistance training volume load and strength. However, no other internal or external training load parameters showed a consistent relationship with physical qualities. The authors attributed the inconsistency in their findings to the complex and multi-factorial nature of the load-response relationship. They emphasized that various factors, including physical qualities, stress, sleep, nutrition, and maturation all play a significant role in influencing an individual’s response to training load during adolescence. While this review included both other sports as well as shorter observation periods, it does help to shed light on this complex issue and warrants further investigation.

Moreover, when comparing the trends for improved physical characteristics observed in this study to those reported in non-athletes [17, 31, 51], as well as athletes in other sports [45, 52], there are some notable distinctions. Specifically, the findings indicate more pronounced yearly improvements in sprint time in team sport athletes (boys 3.6% vs 3.0%, girls 1.9% vs. 1.3%) from 12 to 16 years [17, 31], while development in handgrip strength (boys 11% vs 17%, girls 7.3% vs 7.2%) and jump performance (boys 6.7% vs 11%, girls 4.7% vs 6.6%) [17, 51] were observed to be in non-athletes. Additionally, Philippaerts et al. [55] reported only small differences in the rate of development in various physical characteristics between athletes and non-athletes. These findings further emphasize the uncertain additive effects of training load and sport participation on physical development, to the known effects of growth and maturation. This warrants additional research in this area to better understand the effects of training load on physical development during adolescence. Additionally, factors like training content, intensity, and quality should be explored to better pinpoint which components of training load could promote physical development in young team sport athletes and whether different components are more important in certain phases of adolescence.

### Limitations of the research literature

The present review highlights several gaps in the current literature regarding the development of physical characteristics in adolescent team sport athletes. Firstly, there is a significant underrepresentation of girls in the studies, and even fewer studies investigating longitudinal physical development of girls. This scarcity reduced the available data for analyzing sex differences in physical development and weakens the overall evidence. Even fewer studies have also directly compared boys with girls using the same testing methodology, which could be a good way to directly compare their development. Moreover, the lack of studies focusing on older age groups hampers a thorough exploration of the developmental trajectory during the adolescent period. This knowledge gap poses challenges in designing effective long-term development programs that cater to the unique need of athletes preparing for the senior level.

The lack of maturation data in the majority of studies, and the variations in methodology among those that did incorporate it, made it difficult to conduct a comprehensive analysis on differences in development of physical characteristics among distinct maturity groups. Previous research has indicated that athletes at different maturity stages develop their physical characteristics at different rates [70], but this area remains relatively unexplored and warrants further investigation. Including data on the, potentially large, individual variation in biological age during these years, a more accurate understanding of the influence of maturation on physical characteristics in adolescent team sport athletes could be achieved.

The limited number of longitudinal studies poses challenges in accurately distinguishing actual physical changes from the influence of sample composition for each age group. Longitudinal studies, although with its challenges regarding dropouts and overall lower sample sizes, makes it is possible to conclude with more certainty a cause-and-effect relationship of the development of different physical characteristics observed, compared to cross-sectional studies that only provide a snapshot of data. However, when pooled together, the results from the cross-sectional studies were found to be consistent and comparable to the results from the longitudinal studies (Fig 3). This supports the validity of the overall conclusions, despite the smaller number of included longitudinal studies.

The overall methodological quality rating was moderate (70 ± 13%; S1 Table). Questions 3 and 5 received the lowest mean rating, meaning most of the studies did not justify their sample size and the rate of eligible persons was not possible to determine. Lack of sample size justification could introduce uncertainty into the representativeness of the study population potentially leading to inadequate statistical power for detecting meaningful effects or relationship. This is a greater issue for age groups or tests with low sample sizes and/or few included studies, while individual studies will likely not have a large impact in larger pooled samples. For the longitudinal studies, loss to follow-up was only reported by 4 studies (11%). The lack of this information makes it difficult to generalize the findings, as the findings might not represent the initial study population.

Lastly, the results in this review are also limited by the large diversity in testing methodologies employed in the literature. The wide variety of different tests used to assess different physical characteristics necessitated the selection of specific tests for analysis, reducing the number of suitable studies to 128 out of the initially included total of 176. This approach offered the advantage of obtaining a more manageable dataset for drawing accurate conclusions and comparisons regarding longitudinal physical development. Including too many different tests for one physical characteristic could lead to erroneous conclusions, as certain tests may measure different physical attributes. The lack of test homogeneity hampers meaningful comparisons between studies for researchers and practitioners. To overcome this limitation, using standardized testing batteries [71, 72] would promote greater test homogeneity and enable consistent comparisons.

## Practical applications

The complex interplay of growth, maturation, and physical characteristics in shaping physical development during adolescence highlights the importance of considering sex differences and age-related variations. Coaches must tailor their approaches to account for the specific developmental trajectories of boys and girls. This includes recognizing the slower rates of physiological development typically observed in girls, and in the period following cessation of maturity for boys and implement training programs to target specific physical characteristics providing appropriate stimuli for team sport athletes’ individual needs. It is also important to be aware of the lesser amount of information on girls’ development, especially for older age groups. This scarcity highlights the need for more information over a larger age-span to better understand development throughout the entire adolescent period.

## Conclusion

This review highlighted the progressive improvement in most physical characteristics during adolescence, in both girls and boys in team sports, largely influence by growth and maturation. During early adolescence, the development is stable and rapid but appears to slow down towards late adolescence. Girls have a slower development compared to boys, which can be attributed to the differences in maturation between the sexes, where boys greatly benefit from a greater increase in testosterone and limb length [17]. While the groups in this review were structured by chronological age, exploring development based on biological age would add interesting insights to better understand the role of maturation on physical development in adolescent athletes. Future research is encouraged to include maturation measures to offer more precise insights into the influence of variation in maturation on longitudinal development of physical characteristics in team sport athletes. With only limited studies investigating the effects of training load and team sports participation on long-term physical development, definitively establishing their impact during this period is challenging. To improve our understanding on this topic, future research should incorporate different measures of training load when investigating changes in physical characteristics in adolescent team sport athletes.

## Supporting information

S1 Checklist PRISMA checklist. (DOCX)

S1 Table Quality assessment questions. na: not applicable. cd: cannot determine.
(DOCX)

S2 Table Methodological quality assessment. 1 = yes, 0 = no or unable to determine (where applicable).
(DOCX)

S3 Table Characteristics of the studies included in the review. B: boys. G: girls. CS: cross-sectional. L: longitudinal. ML: mixed-longitudinal. Y: years. CMJ: countermovement jump. YYIR1: yoyo intermittent recovery test level 1. VO_2**-**_max: maximal oxygen uptake. N/A: not available. UMTT: university of montreal track test. SJ: squat jump. CoD: change of direction. AFL: Australian football league. ISO: isometric. IMPT: isometric mid-thigh pull. ISRT: interval shuttle run test. RSA: repeated sprint ability. RDL: romanian dead lift. YYER1: yoyo endurance recovery level 1.PWC: physical work capacity. The competitive level is classified according to the descriptions provided in each respective article.
(DOCX)

S4 Table Number of studies for each age group for each physical characteristic. CMJ: countermovement jump. CoD: change of direction.
(DOCX)

S1 Data Extracted data from all included papers. (XLSX)

S1 Fig Distribution of the studies investigating different team sports included in the review. (TIF)

## List of abbreviations

PRISMA: Preferred Reporting Items for Systematic Reviews and Meta-Analyses
CoD: change of direction
CMJ: countermovement jump
YYIR1: yo-yo intermittent recovery test level 1
ISO: isometric
RM: repetition maximum
PT: peak torque
VO_2_-max: maximal oxygen uptake

## References

[1] **<**References>. Dolci F, Hart NH, Kilding AE, Chivers P, Piggott B, Spiteri T. Physical and Energetic Demand of Soccer: A Brief Review. Strength Cond J. 2020;42(3):70–7.

[2] Stojanović E, Stojiljković N, Scanlan AT, Dalbo VJ, Berkelmans DM, Milanović Z. The Activity Demands and Physiological Responses Encountered During Basketball Match-Play: A Systematic Review. Sports Med. 2018;48(1):111–35. doi: 10.1007/s40279-017-0794-z 29039018

[3] Taylor JB, Wright AA, Dischiavi SL, Townsend MA, Marmon AR. Activity Demands During Multi-Directional Team Sports: A Systematic Review. Sports Med. 2017;47(12):2533–51. doi: 10.1007/s40279-017-0772-5 28801751

[4] Ziv G, Lidor R. Physical characteristics, physiological attributes, and on-court performances of handball players: A review. Eur J Sport Sci. 2009;9(6):375–86.

[5] Loturco I, Bishop C, Freitas TT, Pereira LA, Jeffreys I. Vertical Force Production in Soccer: Mechanical Aspects and Applied Training Strategies. Strength Cond J. 2020;42(2):6–15.

[6] Wen N, Dalbo VJ, Burgos B, Pyne DB, Scanlan AT. Power testing in basketball: current practice and future recommendations. J Strength Cond Res. 2018;32(9):2677–91. doi: 10.1519/JSC.0000000000002459 29401204

[7] Baena-Raya A, Soriano-Maldonado A, Rodríguez-Pérez MA, García-De-Alcaraz A, Ortega-Becerra M, Jiménez-Reyes P, et al. The force-velocity profile as determinant of spike and serve ball speed in top-level male volleyball players. PLoS One. 2021;16(4):249612. doi: 10.1371/journal.pone.0249612 33798256 PMC8018657

[8] Rebelo A, Brito J, Maia J, Coelho-E-Silva MJ, Figueiredo AJ, Bangsbo J, et al. Anthropometric characteristics, physical fitness and technical performance of under-19 soccer players by competitive level and field position. Int J Sports Med. 2013;34(4):312–7. doi: 10.1055/s-0032-1323729 23059558

[9] Saward C, Hulse M, Morris JG, Goto H, Sunderland C, Nevill ME. Longitudinal Physical Development of Future Professional Male Soccer Players: Implications for Talent Identification and Development? Front Sports Act Living. 2020;2. 33345142 10.3389/fspor.2020.578203 PMC7739714

[10] Deprez D, Fransen J, Lenoir M, Philippaerts R, Vaeyens R. A retrospective study on anthropometrical, physical fitness, and motor coordination characteristics that influence dropout, contract status, and first-team playing time in high-level soccer players aged eight to eighteen years. J Strength Cond Res. 2015;29(6):1692–704. doi: 10.1519/JSC.0000000000000806 26010800

[11] Emmonds S, Till K, Jones B, Mellis M, Pears M. Anthropometric, speed and endurance characteristics of English academy soccer players: Do they influence obtaining a professional contract at 18 years of age? Int J Sports Sci Coach. 2016;11(2):212–8.

[12] Aquino R, Alves IS, Padilha MB, Casanova F, Puggina EF, Maia J. Multivariate Profiles of Selected Versus non-Selected Elite Youth Brazilian Soccer Players. J Hum Kinet. 2017;60(1):113–21. doi: 10.1515/hukin-2017-0094 29339991 PMC5765791

[13] Barnes C, Archer DT, Hogg B, Bush M, Bradley PS. The evolution of physical and technical performance parameters in the English premier league. Int J Sports Med. 2014;35(13):1095–100. doi: 10.1055/s-0034-1375695 25009969

[14] Allen T, Taberner M, Zhilkin M, Rhodes D. Running more than before? The evolution of running load demands in the English Premier League. Int J Sports Sci Coach. 2023;

[15] Williams AM, Ford PR, Drust B. Talent identification and development in soccer since the millennium. J Sports Sci. 2020;38(11–12):1199–210. doi: 10.1080/02640414.2020.1766647 32568000

[16] Pyne DB, Spencer M, Mujika I. Improving the value of fitness testing for football. Int J Sports Physiol Perform. 2014;9(3):511–4. doi: 10.1123/ijspp.2013-0453 24231433

[17] Malina RM, Bouchard C, Bar-Or O. Growth, Maturation, and Physical activity. 2nd ed. Champaign, Ill: Human Kinetics; 2004.

[18] Myer GD, Faigenbaum AD, Ford KR, Best TM, Bergeron MF, Hewett TE. When to initiate integrative neuromuscular training to reduce sports-related injuries and enhance health in youth? Curr Sports Med Rep. 2011;10(3):157–66. doi: 10.1249/JSR.0b013e31821b1442 21623307 PMC3105332

[19] Gabbett TJ, Whyte DG, Hartwig TB, Wescombe H, Naughton GA. The relationship between workloads, physical performance, injury and illness in adolescent male football players. Sports Med. 2014;44(7):989–1003. doi: 10.1007/s40279-014-0179-5 24715614

[20] Rice J, Brownlee TE, McRobert AP, Ade J, Drust B, Malone JJ. The association between training load and physical development in professional male youth soccer players: a systematic review. Int J Sports Sci Coach. 2022;17(6):1488–505.

[21] Matos N, Winsley RJ. Trainability of young athletes and overtraining. J Sports Sci Med. 2007;6(3):353–67. 24149422 PMC3787286

[22] Silva AF, Alvurdu S, Akyildiz Z, Badicu G, Greco G, Clemente FM. Variations of the Locomotor Profile, Sprinting, Change-of-Direction, and Jumping Performances in Youth Soccer Players: Interactions between Playing Positions and Age-Groups. Int J Environ Res Public Health. 2022;19(2):998. doi: 10.3390/ijerph19020998 35055819 PMC8775578

[23] Gabbett TJ. Physiological characteristics of junior and senior rugby league players. Br J Sports Med. 2002;36(5):334–9. doi: 10.1136/bjsm.36.5.334 12351330 PMC1724544

[24] Owen C, Till K, Jones B, Weakley J. Testing methods and physical qualities of male age grade rugby union players: A systematic review. PLoS One. 2020;15(6):e0233796. doi: 10.1371/journal.pone.0233796 32497130 PMC7272054

[25] Till K, Cobley S, O’ Hara J, Cooke C, Chapman C. Considering maturation status and relative age in the longitudinal evaluation of junior rugby league players. Scand J Med Sci Sports. 2014;24(3):569–76. doi: 10.1111/sms.12033 23289942

[26] Till K, Cobley S, O’Hara J, Chapman C, Cooke C. A longitudinal evaluation of anthropometric and fitness characteristics in junior rugby league players considering playing position and selection level. J Sci Med Sport. 2013;16(5):438–43. doi: 10.1016/j.jsams.2012.09.002 23072898

[27] Till K, Tester E, Jones B, Emmonds S, Fahey J, Cooke C. Anthropometric and physical characteristics of english academy rugby league players. J Strength Cond Res. 2014;28(2):319–27. doi: 10.1519/JSC.0b013e3182a73c0e 23942164

[28] Jones HE. Motor performance and growth: a developmental study of static dynamometric strength. University of California Press. 1949;1(1).

[29] Round JM, Jones DA, Honour JW, Nevill AM. Hormonal factors in the development of differences in strength between boys and girls during adolescence: A longitudinal study. Ann Hum Biol. 1999;26(1):49–62. doi: 10.1080/030144699282976 9974083

[30] Krahenbuhl G, Skinner J, Kohrt W. Developmental Aspects of Maximal Aerobic Power in Children. Exerc Sport Sci Rev. 1985;13:503–38. 3891374

[31] Greier K, Drenowatz C, Ruedl G, Kirschner W, Mitmannsgruber P, Greier C. Physical Fitness across 11- to 17-Year-Old Adolescents: A Cross-Sectional Study in 2267 Austrian Middle- and High-School Students. Advances in Physical Education. 2019;09(04):258–69.

[32] Liberati A, Altman DG, Tetzlaff J, Mulrow C, Gøtzsche PC, Ioannidis JPA, et al. The PRISMA statement for reporting systematic reviews and meta-analyses of studies that evaluate health care interventions: Explanation and elaboration. PLoS Med. 2009;6(7):e1000100. doi: 10.1371/journal.pmed.1000100 19621070 PMC2707010

[33] Moher D, Liberati A, Tetzlaff J, Altman DG. Preferred reporting items for systematic reviews and meta-analyses: The PRISMA statement. BMJ. 2009;339(7716):332–6. PMC3090117 21603045

[34] Rohatgi A. Webplotdigitizer: Version 4.6. 2022.

[35] Baxter-Jones A, Goldstein H, Helms P. The development of aerobic power in young athletes. J Appl Physiol (1985). 1993;75(3):1160–7. doi: 10.1152/jappl.1993.75.3.1160 8226525

[36] Elferink-Gemser MT, Visscher C, Van Duijn MAJ, Lemmink KAPM. Development of the interval endurance capacity in elite and sub-elite youth field hockey players. Br J Sports Med. 2006;40(4):340–5. doi: 10.1136/bjsm.2005.023044 16556790 PMC2577536

[37] Roescher CR, Elferink-Gemser MT, Huijgen BCH, Visscher C. Soccer endurance development in professionals. Int J Sports Med. 2010;31(3):174–9. doi: 10.1055/s-0029-1243254 20157870

[38] Valente-dos-Santos J, Coelho-e-Silva MJ, Duarte J, Figueiredo AJ, liparotti JR, sherar LB, et al. Longitudinal predictors of aerobic performance in adolescent soccer players. Medicina (Kaunas). 2012;48(8):410–6. 23128461

[39] Armstrong N, van Mechelen W. Oxford Textbook of Children’s Sport and Exercise Medicine. 3rd ed. Oxford University Press; 2017.

[40] Geeson-Brown T, Jones B, Till K, Chantler S, Deighton K. Body composition differences by age and playing standard in male rugby union and rugby league: A systematic review and meta-analysis. J Sports Sci. 2020;38(19):2161–76. doi: 10.1080/02640414.2020.1775990 32546054

[41] Duthie G, Pyne D, Hooper S. Applied Physiology and Game Analysis of Rugby Union. Sports Med. 2003;33(13):973–91. doi: 10.2165/00007256-200333130-00003 14606925

[42] Fragala MS, Kraemer WJ, Denegar CR, Maresh CM, Mastro AM, Volek JS. Neuroendocrine-Immune Interactions and Responses to Exercise. Sports Med. 2012;41(8):621–39. 10.2165/11590430-000000000-00000 21780849

[43] Goswami B, Singha Roy A, Dalui R, Bandyopadhyay A. Impact of Pubertal Growth on Physical Fitness. Am J Sports Sci Med. 2014;2(5A):34–9.

[44] Siervogel RM, Demerath EW, Schubert C, Remsberg KE, Chumlea WC, Sun S, et al. Puberty and body composition. Horm Res. 2003;60(Suppl. 1):36–45. doi: 10.1159/000071224 12955016

[45] Handelsman DJ. Sex differences in athletic performance emerge coinciding with the onset of male puberty. Clin Endocrinol (Oxf). 2017;87(1):68–72. doi: 10.1111/cen.13350 28397355

[46] Weakley JJS, Till K, Darrall-Jones J, Roe GAB, Phibbs PJ, Read DB, et al. Strength and conditioning practices in adolescent rugby players: relationship with changes in physical qualities. J Strength Cond Res. 2017;33(9):2361–9. 10.1519/JSC.0000000000001828 28146030

[47] Buchanan PA, Vardaxis VG. Sex-Related and Age-Related Differences in Knee Strength of Basketball Players Ages 11–17 Years. J Athl Train. 2003;38(3):231–7. 14608433 PMC233177

[48] Emmonds S, Morris R, Murray E, Robinson C, Turner L, Jones B. The influence of age and maturity status on the maximum and explosive strength characteristics of elite youth female soccer players. Sci Med Football. 2017;1(3):209–15.

[49] Emmonds S, Scantlebury S, Murray E, Turner L, Robsinon C, Jones B. Physical Characteristics of Elite Youth Female Soccer Players Characterized by Maturity Status. J Strength Cond Res. 2020;34(8):2321–8. doi: 10.1519/JSC.0000000000002795 30199446

[50] Forbes H, Bullers A, Lovell A, McNaughton LR, Polman RC, Siegler JC. Relative torque profiles of elite male youth footballers: Effects of age and pubertal development. Int J Sports Med. 2009;30(8):592–7. doi: 10.1055/s-0029-1202817 19468968

[51] Ortega FB, Artero EG, Ruiz JR, España-Romero V, Jiménez-Pavón D, Vicente-Rodriguez G, et al. Physical fitness levels among European adolescents: The HELENA study. Br J Sports Med. 2011;45(1):20–9. doi: 10.1136/bjsm.2009.062679 19700434

[52] Tønnessen E, Svendsen IS, Olsen IC, Guttormsen A, Haugen T. Performance development in adolescent track and field athletes according to age, sex and sport discipline. PLoS One. 2015;10(6):e0129014. doi: 10.1371/journal.pone.0129014 26043192 PMC4456243

[53] Mendez-Villanueva A, Buchheit M, Kuitunen S, Douglas A, Peltola E, Bourdon P. Age-related differences in acceleration, maximum running speed, and repeated-sprint performance in young soccer players. J Sports Sci. 2011;29(5):477–84. doi: 10.1080/02640414.2010.536248 21225488

[54] Meyers RW, Oliver JL, Hughes MG, Lloyd RS, Cronin JB. Influence of age, maturity, and body size on the spatiotemporal determinants of maximal sprint speed in boys. J Strength Cond Res. 2017;31(4):1009–16. doi: 10.1519/JSC.0000000000001310 26694506

[55] Philippaerts RM, Vaeyens R, Janssens M, Van Renterghem B, Matthys D, Craen R, et al. The relationship between peak height velocity and physical performance in youth soccer players. J Sports Sci. 2006;24(3):221–30. doi: 10.1080/02640410500189371 16368632

[56] Schepens B., Willems P. A., Cavagna G. A. The mechanics of running in children. J Physiol. 1998;509(3):927–40. doi: 10.1111/j.1469-7793.1998.927bm.x 9596810 PMC2231007

[57] Hunter JP, Marshall RN, McNair PJ. Interaction of Step Length and Step Rate during Sprint Running. Med Sci Sports Exerc. 2004;36(2):261–71. doi: 10.1249/01.MSS.0000113664.15777.53 14767249

[58] Lloyd RS, Oliver JL. The Youth Physical Development Model: A New Approach to Long-Term Athletic Development. Strength Cond J. 2012;34(3):61–72.

[59] Suchomel TJ, Nimphius S, Stone MH. The Importance of Muscular Strength in Athletic Performance. Sports Med. 2016;46(10):1419–49. doi: 10.1007/s40279-016-0486-0 26838985

[60] Young WB, Dawson B, Henry GJ. Agility and change-of-direction speed are independent skills: Implications for training for agility in invasion sports. Int J Sports Sci Coach. 2015;10(1):159–69.

[61] Bangsbo J, Iaia FM, Krustrup P. The Yo-Yo intermittent recovery test: A useful tool for evaluation of physical performance in intermittent sports. Sports Med. 2008;38(1):37–51. doi: 10.2165/00007256-200838010-00004 18081366

[62] Krustrup P, Mohr M, Amstrup T, Rysgaard T, Johansen J, Steensberg A, et al. The Yo-Yo intermittent recovery test: Physiological response, reliability, and validity. Med Sci Sports Exerc. 2003;35(4):697–705. doi: 10.1249/01.MSS.0000058441.94520.32 12673156

[63] Leppänen M, Uotila A, Tokola K, Forsman-Lampinen H, Kujala UM, Parkkari J, et al. Players with high physical fitness are at greater risk of injury in youth football. Scand J Med Sci Sports. 2022;32(11):1625–38. doi: 10.1111/sms.14199 35621388

[64] Landgraff HW, Riiser A, Lihagen M, Skei M, Leirstein S, Hallén J. Longitudinal changes in maximal oxygen uptake in adolescent girls and boys with different training backgrounds. Scand J Med Sci Sports. 2021;31(S1):65–72. doi: 10.1111/sms.13765 33871085

[65] Kemper HCG, Dekker HJP, Ootjers MG, Post B, Snel J, Splinter PG, et al. Growth and health of teenagers in the Netherlands: Survey of Multidisciplinary Longitudinal Studies and Comparison to Recent Results of a Dutch Study. Int J Sports Med. 1983;4(4):202–14. doi: 10.1055/s-2008-1026036 6654545

[66] Armstrong N, Williams J, Balding J, Gentle P, Kirby B. The peak oxygen uptake of British children with reference to age, sex and sexual maturity. Eur J Appl Physiol Occup Physiol. 1991;62(5):369–75. doi: 10.1007/BF00634975 1874245

[67] Wrigley RD, Drust B, Stratton G, Atkinson G, Gregson W. Long-term soccer-specific training enhances the rate of physical development of academy soccer players independent of maturation status. Int J Sports Med. 20140710th ed. 2014;35(13):1090–4. doi: 10.1055/s-0034-1375616 25009972

[68] Bucher Sandbakk S, Walther J, Solli GS, Tønnessen E, Haugen T. Training Quality-What Is It and How Can We Improve It? Int J Sports Physiol Perform. 2023;18(5):557–60. doi: 10.1123/ijspp.2022-0484 36965489

[69] Dudley C, Johnston R, Jones B, Till K, Westbrook H, Weakley J. Methods of Monitoring Internal and External Loads and Their Relationships with Physical Qualities, Injury, or Illness in Adolescent Athletes: A Systematic Review and Best-Evidence Synthesis. Sports Med. 2023 Aug 1;53(8):1559–93. doi: 10.1007/s40279-023-01844-x 37071283 PMC10356657

[70] Deprez D, Buchheit M, Fransen J, Pion J, Lenoir M, Philippaerts RM, et al. A Longitudinal Study Investigating the Stability of Anthropometry and Soccer-Specific Endurance in Pubertal High-Level Youth Soccer Players [Internet]. Vol. 14, ©Journal of Sports Science and Medicine. 2015. Available from: http://www.jssm.org PMC4424473 25983593

[71] Till K, Scantlebury S, Jones B. Anthropometric and Physical Qualities of Elite Male Youth Rugby League Players. Sports Med. 2017;47(11):2171–86. doi: 10.1007/s40279-017-0745-8 28578541 PMC5633637

[72] Till K, Collins N, Mccormack S, Owen C, Weaving D, Jones B. Challenges and Solutions for Physical Testing in Sport: The Profiling Physical Qualities Tool. Strength Cond J. 2023;45(1):29–39.

