# Supplementary material for: The development of physical characteristics in adolescent team sport athletes: A systematic review
Source: PLoS One. 2023 Dec 21;18(12):e0296181. doi: 10.1371/journal.pone.0296181 (PMC10735042; doi:10.1371/journal.pone.0296181)
Supplement: S1 Table — na: not applicable. cd: cannot determine. (DOCX) [file pone.0296181.s002.docx]

| **Question** | | **Answer** | **Score** |
| --- | --- | --- | --- |
| Q1 | Was the research question or objective in this paper clearly stated? | Yes = 1, No/na/cd = 0 | 0-1 |
| Q2 | Was the study population clearly specified and defined? | Yes = 1, No/na/cd = 0 | 0-1 |
| Q3 | Was the participation rate of eligible persons at least 50%? | Yes = 1, No/na/cd = 0 | 0-1 |
| Q4 | Were all the subjects selected or recruited from the same or similar populations (including the same time period)? Were inclusion and exclusion criteria for being in the study prespecified and applied uniformly to all participants? | Yes = 1, No/na/cd = 0 | 0-1 |
| Q5 | Was a sample size justification, power description, or variance and effect estimates provided? | Yes = 1, No/na/cd = 0 | 0-1 |
| Q7 | Was the timeframe sufficient so that one could reasonably expect to see an association between exposure and outcome if it existed? | Yes = 1, No/na/cd = 0 | 0-1 |
| Q9 | Were the exposure measures (independent variables) clearly defined, valid, reliable, and implemented consistently across all study participants? | Yes = 1, No/na/cd = 0 | 0-1 |
| Q11 | Were the outcome measures (dependent variables) clearly defined, valid, reliable, and implemented consistently across all study participants? | Yes = 1, No/na/cd = 0 | 0-1 |
| Q13 | Was loss to follow-up after baseline 20% or less? | Yes = 1, No/na/cd = 0 | 0-1 |
