# Supplementary material for: The development of physical characteristics in adolescent team sport athletes: A systematic review
Source: PLoS One. 2023 Dec 21;18(12):e0296181. doi: 10.1371/journal.pone.0296181 (PMC10735042; doi:10.1371/journal.pone.0296181)
Supplement: S2 Table — 1 = yes, 0 = no or unable to determine (where applicable). (DOCX) [file pone.0296181.s003.docx]

## **Supplementary Table S2:**

**Table 4** methodological quality assessment

| **Study** | **Question number** | | | | | | | | | **Total score (out of max)** |
| --- | --- | --- | --- | --- | --- | --- | --- | --- | --- | --- |
|  | **1** | **2** | **3** | **4** | **5** | **7** | **9** | **11** | **13** |  |
| Agrebi et al. (73) | 1 | 1 | 0 | 1 | 0 | 0 | 1 | 1 | 0 | 5/7 |
| Alvurdu et al. (74) | 1 | 1 | 0 | 1 | 0 | 0 | 0 | 1 | 0 | 4/7 |
| Andrade et al. (75) | 1 | 1 | 0 | 1 | 0 | 0 | 1 | 1 | 0 | 5/7 |
| Andrade et al. (76) | 1 | 1 | 0 | 1 | 0 | 0 | 1 | 1 | 0 | 5/7 |
| Atan et al. (77) | 1 | 1 | 0 | 1 | 0 | 0 | 0 | 1 | 0 | 4/7 |
| Barr et al. (78) | 1 | 1 | 0 | 0 | 0 | 1 | 1 | 1 | 0 | 5/9 |
| Baxter-Jones et al. (35) | 1 | 1 | 0 | 0 | 0 | 1 | 1 | 1 | 0 | 5/7 |
| Bennett et al. (79) | 1 | 1 | 0 | 1 | 0 | 0 | 1 | 1 | 0 | 5/9 |
| Bidaurrazaga et al. (80) | 1 | 1 | 0 | 1 | 0 | 1 | 1 | 1 | 0 | 6/7 |
| Bona et al. (81) | 1 | 1 | 0 | 1 | 0 | 0 | 1 | 1 | 0 | 5/7 |
| Brent et al. (82) | 1 | 1 | 0 | 1 | 0 | 1 | 0 | 1 | 0 | 5/7 |
| Buchanan et al. (47) | 1 | 1 | 0 | 1 | 0 | 0 | 1 | 1 | 0 | 5/7 |
| Buchheit et al. (83) | 1 | 1 | 0 | 1 | 0 | 0 | 1 | 1 | 0 | 5/7 |
| Byrne et al. (84) | 1 | 1 | 0 | 1 | 0 | 0 | 1 | 1 | 0 | 5/7 |
| Cardosa de Arajuo et al. (85) | 1 | 1 | 0 | 1 | 0 | 0 | 1 | 1 | 0 | 5/7 |
| Carvalho et al. (86) | 1 | 1 | 0 | 1 | 0 | 1 | 1 | 1 | 0 | 6/7 |
| Carvalho et al. (87) | 1 | 1 | 0 | 1 | 0 | 0 | 1 | 1 | 0 | 5/7 |
| Carvalho et al. (88) | 1 | 1 | 0 | 1 | 0 | 1 | 1 | 1 | 0 | 6/7 |
| Carvalho et al. (89) | 1 | 1 | 0 | 1 | 0 | 1 | 0 | 1 | 0 | 5/7 |
| Chiwaridzo et al. (90) | 1 | 1 | 0 | 1 | 0 | 0 | 1 | 1 | 0 | 5/7 |
| Ciacci et al. (91) | 1 | 1 | 0 | 1 | 0 | 0 | 1 | 1 | 0 | 5/7 |
| Cobley et al. (92) | 1 | 1 | 0 | 1 | 0 | 1 | 1 | 1 | 0 | 6/7 |
| Condello et al. (93) | 1 | 1 | 0 | 1 | 0 | 0 | 0 | 1 | 0 | 4/7 |
| Cordingley et al. (94) | 1 | 1 | 0 | 1 | 0 | 1 | 0 | 1 | 0 | 5/7 |
| Coutinho et al. (95) | 1 | 1 | 0 | 1 | 0 | 0 | 1 | 1 | 0 | 5/7 |
| Craig et al. (96) | 1 | 1 | 1 | 1 | 0 | 1 | 0 | 1 | 0 | 6/9 |
| Cripps et al. (97) | 1 | 1 | 0 | 1 | 0 | 1 | 1 | 1 | 0 | 6/7 |
| Cunha et al. (98) | 1 | 1 | 0 | 1 | 0 | 0 | 1 | 1 | 0 | 5/7 |
| Degache et al. (99) | 1 | 1 | 0 | 1 | 0 | 0 | 0 | 1 | 0 | 4/9 |
| DeLang et al. (100) | 1 | 1 | 0 | 1 | 0 | 0 | 1 | 1 | 0 | 5/7 |
| Deprez et al. (101) | 1 | 1 | 0 | 1 | 0 | 1 | 1 | 1 | 0 | 6/7 |
| Deprez et al. (102) | 1 | 1 | 0 | 1 | 0 | 1 | 1 | 1 | 0 | 6/7 |
| Deprez et al. (103) | 1 | 1 | 0 | 1 | 0 | 1 | 1 | 1 | 0 | 6/7 |
| Deprez et al. (104) | 1 | 1 | 0 | 1 | 0 | 1 | 1 | 1 | 0 | 6/7 |
| Deprez et al. (105) | 1 | 1 | 0 | 1 | 0 | 0 | 1 | 1 | 0 | 5/7 |
| Deprez et al. (10) | 1 | 1 | 0 | 1 | 0 | 1 | 1 | 1 | 0 | 6/7 |
| **Study** | **Question number** | | | | | | | | | **Total score (out of max)** |
|  | **1** | **2** | **3** | **4** | **5** | **7** | **9** | **11** | **13** |  |
| Deprez et al. (106) | 1 | 1 | 0 | 1 | 0 | 1 | 1 | 1 | 0 | 6/7 |
| Dobbs et al. (107) | 1 | 1 | 0 | 1 | 0 | 0 | 0 | 1 | 0 | 4/7 |
| Duarte et al. (108) | 1 | 1 | 0 | 1 | 0 | 0 | 1 | 1 | 0 | 5/7 |
| Duarte et al. (109) | 1 | 1 | 0 | 1 | 0 | 0 | 1 | 1 | 0 | 5/7 |
| Dugdale et al. (110) | 1 | 1 | 0 | 1 | 0 | 1 | 0 | 1 | 0 | 5/7 |
| Elferink-Gemser et al. (36) | 1 | 1 | 0 | 0 | 0 | 0 | 1 | 1 | 0 | 4/7 |
| Elferink-Gemser et al. (111) | 1 | 1 | 0 | 1 | 0 | 1 | 1 | 1 | 0 | 6/7 |
| Emmonds et al. (112) | 1 | 1 | 0 | 1 | 0 | 1 | 1 | 1 | 0 | 6/7 |
| Emmonds et al. (49) | 1 | 1 | 0 | 1 | 0 | 0 | 1 | 1 | 0 | 5/7 |
| Eskandarifard et al. (113) | 1 | 1 | 0 | 1 | 0 | 0 | 1 | 1 | 0 | 5/9 |
| Faltstrom et al. (114) | 1 | 1 | 0 | 1 | 0 | 0 | 0 | 1 | 0 | 4/7 |
| Fernandez-Galvan et al. (115) | 1 | 1 | 0 | 1 | 1 | 0 | 1 | 1 | 0 | 6/7 |
| Firolli et al. (116) | 1 | 1 | 0 | 1 | 0 | 0 | 0 | 1 | 0 | 4/7 |
| Forbes et al. (117) | 1 | 1 | 0 | 1 | 0 | 0 | 1 | 1 | 0 | 5/7 |
| Francini et al. (118) | 1 | 1 | 0 | 1 | 0 | 0 | 1 | 1 | 0 | 5/7 |
| Gabbett et al. (119) | 1 | 1 | 0 | 1 | 0 | 0 | 1 | 1 | 0 | 5/7 |
| Gastin et al. (120) | 1 | 1 | 0 | 1 | 0 | 0 | 1 | 1 | 0 | 5/7 |
| Gaudion et al. (121) | 1 | 1 | 0 | 1 | 0 | 0 | 0 | 1 | 0 | 4/7 |
| Giovanni et al. | 1 | 1 | 0 | 1 | 0 | 1 | 1 | 1 | 0 | 6/7 |
| Giminiani et al. (122) | 1 | 1 | 0 | 1 | 0 | 0 | 1 | 1 | 0 | 5/7 |
| Gonaus et al. (123) | 1 | 1 | 0 | 1 | 0 | 1 | 0 | 1 | 0 | 5/9 |
| Guimaraes et al. (124) | 1 | 1 | 0 | 1 | 0 | 0 | 1 | 1 | 0 | 5/7 |
| Guimaraes et al. (125) | 1 | 1 | 0 | 1 | 0 | 0 | 1 | 1 | 0 | 5/7 |
| Hamilton et al. (126) | 1 | 1 | 0 | 1 | 0 | 0 | 0 | 1 | 0 | 4/7 |
| Hammami et al. (127) | 1 | 1 | 0 | 1 | 0 | 0 | 1 | 1 | 0 | 5/7 |
| Hammami et al. (128) | 1 | 1 | 0 | 1 | 0 | 0 | 1 | 1 | 0 | 5/7 |
| Hansen et al. (129) | 1 | 1 | 0 | 1 | 0 | 1 | 1 | 1 | 0 | 6/7 |
| Hansen et al. (130) | 1 | 1 | 0 | 1 | 0 | 1 | 1 | 1 | 0 | 6/7 |
| Haycraft et al. (131) | 1 | 1 | 0 | 1 | 0 | 0 | 1 | 1 | 0 | 5/7 |
| Hirose et al. (132) | 1 | 1 | 0 | 1 | 0 | 1 | 1 | 1 | 0 | 6/7 |
| Hirose et al. (133) | 1 | 1 | 0 | 1 | 0 | 0 | 1 | 1 | 0 | 5/9 |
| Holm et al. (134) | 1 | 1 | 0 | 1 | 0 | 1 | 1 | 1 | 0 | 6/7 |
| Höner et al. (135) | 1 | 1 | 1 | 1 | 0 | 0 | 0 | 1 | 0 | 5/9 |
| Hoshikawa et al. (136) | 1 | 1 | 0 | 1 | 0 | 0 | 0 | 1 | 0 | 4/9 |
| Huijgen et al. (137) | 1 | 1 | 0 | 1 | 0 | 1 | 1 | 1 | 0 | 6/7 |
| Ishøi et al. (138) | 1 | 1 | 0 | 1 | 0 | 0 | 1 | 1 | 0 | 5/7 |
| Jones et al. (139) | 1 | 1 | 0 | 1 | 0 | 0 | 1 | 1 | 0 | 5/9 |
| Jorge et al. (140) | 1 | 1 | 0 | 1 | 0 | 0 | 1 | 1 | 0 | 5/7 |
| Keiner et al. (141) | 1 | 1 | 0 | 1 | 0 | 1 | 1 | 1 | 1 | 7/7 |
| **Study** | **Question number** | | | | | | | | | **Total score (out of max)** |
|  | **1** | **2** | **3** | **4** | **5** | **7** | **9** | **11** | **13** |  |
| Keiner et al. (142) | 1 | 1 | 0 | 1 | 0 | 1 | 0 | 1 | 0 | 5/7 |
| Keiner et al. (143) | 1 | 1 | 0 | 1 | 0 | 0 | 0 | 1 | 0 | 4/7 |
| Keiner et al. (144) | 1 | 1 | 0 | 1 | 0 | 0 | 1 | 1 | 0 | 5/7 |
| Keiner et al. (145) | 1 | 1 | 0 | 1 | 0 | 1 | 0 | 1 | 0 | 5/9 |
| Kelly et al. (146) | 1 | 1 | 0 | 1 | 0 | 0 | 1 | 1 | 0 | 5/9 |
| Knoop et al. (147) | 1 | 1 | 0 | 1 | 0 | 0 | 1 | 1 | 0 | 5/7 |
| Kobal et al. (148) | 1 | 1 | 0 | 1 | 0 | 0 | 1 | 1 | 0 | 5/7 |
| Kokinda et al. (149) | 1 | 1 | 0 | 1 | 0 | 0 | 1 | 1 | 0 | 5/9 |
| Konieczna et al. (150) | 1 | 1 | 0 | 1 | 0 | 1 | 1 | 1 | 0 | 6/7 |
| Koryahin et al. (151) | 1 | 1 | 0 | 1 | 0 | 0 | 0 | 1 | 0 | 4/7 |
| Kos et al. (152) | 1 | 1 | 0 | 1 | 0 | 0 | 1 | 1 | 0 | 5/7 |
| Kruse et al. (153) | 1 | 1 | 0 | 1 | 0 | 0 | 0 | 1 | 0 | 4/7 |
| Landgraff et al. (64) | 1 | 1 | 0 | 0 | 0 | 1 | 1 | 1 | 0 | 5/9 |
| Leao et al. (154) | 1 | 1 | 0 | 1 | 0 | 0 | 0 | 0 | 0 | 3/7 |
| Lehnert et al. (155) | 1 | 1 | 0 | 1 | 0 | 0 | 0 | 1 | 0 | 4/7 |
| Leiter et al. (156) | 1 | 1 | 0 | 1 | 0 | 1 | 1 | 1 | 0 | 6/7 |
| Lemos et al. (157) | 1 | 1 | 0 | 1 | 0 | 0 | 0 | 1 | 0 | 4/9 |
| Leppanen et al. (63) | 1 | 1 | 0 | 1 | 0 | 1 | 0 | 1 | 0 | 5/7 |
| Letter et al. (158) | 1 | 1 | 0 | 1 | 0 | 0 | 0 | 1 | 0 | 4/7 |
| Leyhr et al. (159) | 1 | 1 | 0 | 1 | 0 | 1 | 0 | 1 | 0 | 5/7 |
| Leyhr et al. (160) | 1 | 1 | 0 | 1 | 0 | 0 | 0 | 1 | 0 | 4/7 |
| Lima et al. (161) | 1 | 1 | 0 | 1 | 0 | 0 | 1 | 1 | 0 | 5/7 |
| Lolli et al. (162) | 1 | 1 | 0 | 1 | 1 | 0 | 0 | 1 | 0 | 5/9 |
| Loturco et al. (163) | 1 | 1 | 0 | 1 | 0 | 0 | 1 | 1 | 0 | 5/9 |
| Łuszczyk et al. (164) | 1 | 1 | 0 | 1 | 0 | 1 | 1 | 1 | 0 | 6/7 |
| Markovic et al. (165) | 1 | 1 | 0 | 1 | 0 | 0 | 0 | 1 | 0 | 4/7 |
| Markovic et al.(166) | 1 | 1 | 0 | 1 | 0 | 0 | 0 | 1 | 0 | 4/7 |
| Martins et al. (167) | 1 | 1 | 0 | 1 | 1 | 0 | 1 | 1 | 0 | 6/7 |
| Matthys et al. (168) | 1 | 1 | 0 | 1 | 0 | 1 | 1 | 1 | 0 | 6/7 |
| Matthys et al. (169) | 1 | 1 | 0 | 1 | 0 | 0 | 1 | 1 | 0 | 5/9 |
| Mendez-Villanueva et al. (170) | 1 | 1 | 0 | 1 | 0 | 0 | 1 | 1 | 0 | 5/9 |
| Mendez-Villanueva et al. (53) | 1 | 1 | 0 | 1 | 0 | 0 | 1 | 1 | 0 | 5/7 |
| Metaxas et al. (171) | 1 | 1 | 0 | 1 | 0 | 0 | 1 | 1 | 0 | 5/7 |
| Metaxas et al. (172) | 1 | 1 | 0 | 0 | 0 | 0 | 1 | 1 | 0 | 4/7 |
| Mirkov et al. (173) | 1 | 1 | 0 | 1 | 0 | 1 | 0 | 1 | 0 | 5/7 |
| Morris et al. (174) | 1 | 1 | 0 | 1 | 0 | 0 | 1 | 1 | 0 | 5/9 |
| Nedeljkoic et al. (175) | 1 | 1 | 0 | 1 | 0 | 0 | 0 | 1 | 0 | 4/7 |
| Nguyen et al. (176) | 1 | 1 | 0 | 1 | 0 | 1 | 1 | 1 | 0 | 6/7 |
| Niederer et al. (177) | 1 | 1 | 0 | 1 | 0 | 0 | 0 | 1 | 0 | 4/7 |
| **Study** | **Question number** | | | | | | | | | **Total score (out of max)** |
|  | **1** | **2** | **3** | **4** | **5** | **7** | **9** | **11** | **13** |  |
| Nikolaidis et al. (178) | 1 | 1 | 0 | 1 | 0 | 0 | 1 | 1 | 0 | 5/7 |
| Nikolaidis et al. (179) | 1 | 1 | 0 | 1 | 0 | 0 | 1 | 1 | 0 | 5/7 |
| Noon et al. (180) | 1 | 1 | 0 | 1 | 0 | 0 | 1 | 1 | 0 | 5/9 |
| Nutt et al. (181) | 1 | 1 | 0 | 1 | 0 | 0 | 1 | 0 | 0 | 4/7 |
| Nutton et al. (182) | 1 | 1 | 0 | 1 | 0 | 0 | 0 | 1 | 0 | 4/7 |
| Parpa et al. (183) | 1 | 1 | 0 | 1 | 0 | 0 | 0 | 0 | 0 | 3/7 |
| Peek et al. (184) | 1 | 1 | 0 | 1 | 0 | 0 | 0 | 1 | 0 | 4/9 |
| Perroni et al. (185) | 1 | 1 | 0 | 1 | 0 | 0 | 1 | 1 | 0 | 5/7 |
| Petridis et al. (186) | 1 | 1 | 0 | 1 | 0 | 0 | 1 | 1 | 0 | 5/7 |
| Pizzigalli et al. (187) | 1 | 1 | 0 | 1 | 0 | 0 | 0 | 1 | 0 | 4/7 |
| Quatman et al. (188) | 1 | 1 | 0 | 1 | 0 | 1 | 1 | 1 | 0 | 6/7 |
| Read et al. (189) | 1 | 1 | 0 | 1 | 0 | 0 | 1 | 1 | 0 | 5/7 |
| Read et al. (190) | 1 | 1 | 0 | 1 | 1 | 0 | 1 | 1 | 0 | 6/7 |
| Rebelo-Goncalves et al. (191) | 1 | 1 | 0 | 1 | 0 | 1 | 1 | 1 | 0 | 6/7 |
| Reinikainen et al. (192) | 1 | 1 | 0 | 1 | 0 | 1 | 1 | 1 | 0 | 6/7 |
| Ritsche et al. (193) | 1 | 1 | 0 | 1 | 0 | 0 | 1 | 1 | 0 | 5/7 |
| Roe et al. (194) | 1 | 1 | 0 | 1 | 0 | 0 | 0 | 1 | 0 | 4/7 |
| Roe et al. (195) | 1 | 1 | 0 | 1 | 0 | 0 | 1 | 1 | 0 | 5/7 |
| Roescher et al. (37) | 1 | 1 | 0 | 1 | 0 | 1 | 0 | 1 | 0 | 5/7 |
| Salinero et al. (196) | 1 | 1 | 0 | 1 | 0 | 0 | 1 | 1 | 0 | 5/7 |
| Sander et al. (197) | 1 | 1 | 0 | 1 | 0 | 1 | 0 | 1 | 0 | 5/9 |
| Saward et al. (9) | 1 | 1 | 0 | 1 | 0 | 1 | 0 | 1 | 0 | 5/7 |
| Sekine et al. (198) | 1 | 1 | 0 | 1 | 0 | 1 | 1 | 1 | 0 | 6/7 |
| Silva et al. (199) | 1 | 1 | 0 | 1 | 0 | 1 | 1 | 1 | 0 | 6/7 |
| Śliwowski et al. (200) | 1 | 1 | 0 | 1 | 0 | 1 | 1 | 1 | 0 | 6/7 |
| Söderman et al. (201) | 1 | 1 | 0 | 1 | 0 | 0 | 1 | 1 | 0 | 5/7 |
| Spencer et al. (202) | 1 | 1 | 0 | 1 | 0 | 0 | 0 | 1 | 0 | 4/7 |
| Stojomenovic et al. (203) | 1 | 1 | 0 | 1 | 0 | 1 | 1 | 1 | 0 | 6/9 |
| Sugimoto et al. (204) | 1 | 1 | 0 | 0 | 0 | 0 | 0 | 1 | 0 | 3/7 |
| Saavedra et al. (205) | 1 | 1 | 0 | 1 | 0 | 0 | 1 | 1 | 0 | 5/7 |
| Teixeira et al. (206) | 1 | 1 | 0 | 1 | 0 | 0 | 1 | 1 | 0 | 5/7 |
| Te Wierike et al. (207) | 1 | 1 | 0 | 1 | 0 | 1 | 1 | 1 | 0 | 6/9 |
| Till et al. (26) | 1 | 1 | 0 | 1 | 0 | 1 | 1 | 1 | 1 | 7/7 |
| Till et al. (208) | 1 | 1 | 0 | 1 | 0 | 1 | 1 | 1 | 0 | 6/9 |
| Till et al. (25) | 1 | 1 | 0 | 1 | 0 | 1 | 0 | 1 | 0 | 5/7 |
| Till et al. (27) | 1 | 1 | 0 | 1 | 0 | 1 | 0 | 1 | 0 | 5/7 |
| Till et al. (209) | 1 | 1 | 0 | 1 | 0 | 1 | 0 | 1 | 1 | 6/9 |
| Till et al. (210) | 1 | 1 | 0 | 1 | 0 | 1 | 1 | 1 | 1 | 7/7 |
| Till et al. (211) | 1 | 1 | 0 | 1 | 0 | 1 | 1 | 1 | 0 | 6/7 |
| **Study** | **Question number** | | | | | | | | | **Total score (out of max)** |
|  | **1** | **2** | **3** | **4** | **5** | **7** | **9** | **11** | **13** |  |
| Till et al. (212) | 1 | 1 | 0 | 1 | 0 | 0 | 1 | 1 | 0 | 5/9 |
| Till et al. (213) | 1 | 1 | 0 | 1 | 0 | 1 | 1 | 1 | 0 | 6/7 |
| Till et al. (214) | 1 | 1 | 0 | 1 | 0 | 0 | 0 | 1 | 0 | 4/7 |
| Toong et al. (215) | 1 | 1 | 0 | 1 | 0 | 0 | 1 | 1 | 0 | 5/7 |
| Tribolet et al. (216) | 1 | 1 | 0 | 1 | 0 | 1 | 0 | 1 | 0 | 5/9 |
| Vaeyens et al. (217) | 1 | 1 | 0 | 1 | 0 | 1 | 1 | 1 | 0 | 6/7 |
| Valente-dos-Santos et al. (218) | 1 | 1 | 0 | 1 | 0 | 1 | 1 | 1 | 0 | 6/9 |
| Valente-dos-Santos et al. (219) | 1 | 1 | 0 | 1 | 0 | 0 | 1 | 1 | 0 | 5/9 |
| Valente-dos-Santos et al. (38) | 1 | 1 | 0 | 1 | 0 | 1 | 1 | 1 | 0 | 6/7 |
| Valente-dos-Santos et al. (220) | 1 | 1 | 0 | 1 | 0 | 1 | 1 | 1 | 0 | 6/7 |
| Vandendriessche et al. (221) | 1 | 1 | 0 | 1 | 0 | 0 | 1 | 1 | 0 | 5/9 |
| Vargas et al. (222) | 1 | 1 | 0 | 1 | 0 | 0 | 1 | 1 | 0 | 5/7 |
| Vera Assaoka et al. (223) | 1 | 1 | 0 | 1 | 1 | 0 | 1 | 1 | 0 | 6/7 |
| Vernillo et al. (224) | 1 | 1 | 0 | 1 | 0 | 0 | 0 | 1 | 0 | 4/9 |
| Visnapuu et al. (225) | 1 | 1 | 0 | 1 | 0 | 0 | 0 | 1 | 0 | 4/7 |
| Waldron et al. (226) | 1 | 1 | 0 | 1 | 0 | 1 | 1 | 1 | 0 | 6/7 |
| Waldron et al. (227) | 1 | 1 | 0 | 1 | 0 | 1 | 1 | 1 | 0 | 6/7 |
| Williams et al. (228) | 1 | 1 | 0 | 1 | 0 | 1 | 0 | 1 | 0 | 5/9 |
| Wollin et al. (229) | 1 | 1 | 0 | 1 | 0 | 1 | 1 | 1 | 0 | 6/7 |
| Woodcock et al. (230) | 1 | 1 | 0 | 1 | 0 | 1 | 0 | 1 | 0 | 5/7 |
| Woods et al. (231) | 1 | 1 | 0 | 1 | 0 | 0 | 1 | 1 | 0 | 5/7 |
| Wright et al. (232) | 1 | 1 | 0 | 1 | 0 | 1 | 1 | 1 | 0 | 6/7 |
| Yang et al. (233) | 1 | 1 | 0 | 1 | 0 | 0 | 1 | 1 | 0 | 5/7 |
