# Supplementary material for: The development of physical characteristics in adolescent team sport athletes: A systematic review
Source: PLoS One. 2023 Dec 21;18(12):e0296181. doi: 10.1371/journal.pone.0296181 (PMC10735042; doi:10.1371/journal.pone.0296181)
Supplement: S3 Table — B: boys. G: girls. CS: cross-sectional. L: longitudinal. ML: mixed-longitudinal. Y: years. CMJ: countermovement jump. YYIR1: yoyo intermittent recovery test level 1. VO2-max: maximal oxygen uptake. N/A: not available. UMTT: university of montreal track test. SJ: squat jump. CoD: change of direction. AFL: Australian football league. ISO: isometric. IMPT: isometric mid-thigh pull. ISRT: interval shuttle run test. RSA: repeated sprint ability. RDL: romanian dead lift. YYER1: yoyo endurance recovery level 1.PWC: physical work capacity. The competitive level is classified according to the descriptions provided in each respective article. (DOCX) [file pone.0296181.s004.docx]

## **Supplementary Table S3**

**Table 5** Characteristics of the studies included in the review.

| **Study** | **Design, Duration** | **B  (n)** | **G (n)** | **Age (yrs)** | **Competitive level, sport** | **Tests used** |
| --- | --- | --- | --- | --- | --- | --- |
| Agrebi et al. (1) | CS | 24 | 0 | 12\| 16 | National, Handball | Graded bike protocol, specific field test |
| Alvurdu et al. (2) | CS | 68 | 0 | U15\| U16\| U17\| U19 | Turkish super league, Football | 30 m, CMJ, YYIR1 |
| Andrade et al. (3) | CS | 0 | 93 | 12\| 14\| 16 | National, Football | Isokinetic peak torque flexion & extension |
| Andrade et al. (4) | CS | 52 | 33 | 14\| 16\| 17\| 19 | Handball | Peak torque conc external rotator 60*/s |
| Atan et al. (5) | CS | 85 | 0 | 13\| 14\| 15 | Professional academy, Football | 10 m flying, YYIR1 |
| Barr et al. (6) | L, 2 y | 36 | 0 | 19\| 20\| 21 | Rugby | 40 m max speed (m/s) |
| Baxter-Jones et al. (7) | ML, 3 y | 117 | 0 | 13\| 14\| 16 | N/A, Football | VO_2_**_-_**max |
| Bennett et al. (8) | L, 2 y | 412 | 0 | U17\| U18 | State, Football | CMJ |
| Bidaurrazaga et al. (9) | ML, 4 y | 196 | 0 | 12 \| 13 \| 14 | Professional academy, Football | 15 m, CMJ arms, Modified Barrow zigzag |
| Bona et al. (10) | CS | 49 | 0 | 15\| 17 | Provincial first division, Football | Isokinetic peak torque flexion & extension |
| Brent et al. (11) | CS | 79 | 272 | 12\| 13\| 14\| 15\| 16\| 17\| 18 | Football | Isokinetic hip abduction |
| Buchanan et al. (12) | CS | 19 | 22 | 11\| 12\| 13\| 15\| 16\| 17 | Elite, Basketball | Isokinetic peak torque flexion & extension |
| Buchheit et al. (13) | CS | 160 | 0 | 13\| 14\| 15\| 16\| 17 | Elite, Football | 10 m, CMJ, UMTT |
| Byrne et al. (14) | CS | 198 | 113 | 14\| 15\| 16\| 17\| 14\| 15\| 16\| 17 | N/A, Hurling, camogie, Gaelic football | 15 m, CMJ, broad jump, agility 505, modified cooper test, 3 kg medicine ball throw |
| Cardosa de Arajuo et al. (15) | CS | 48 | 0 | 15\| 17\| 20 | Elite, Football | 10 m, 20 m, 30 m, 5 m, 22 m nonlinear sprint |
| Carvalho et al. (16) | ML | 33 | 0 | 12\| 13\| 14 | Professional academy, Football | YYIR1 |
| Carvalho et al. (17) | CS | 0 | 0 | Pre-PHV\| Mid-PHV\| Post-PHV | State level, Basketball | Line drill |
| Carvalho et al. (18) | ML, 4 y | 137 | 0 | 12\| 13\| 14\| 15\| 16 | Professional academy, Football | 15 m max speed, CMJ, modified barrow zig zag, YYIR1 |
| Carvalho et al. (19) | ML, | 0 | 38 | 12\| 13 \| 14 \| 15 \|16 | Basketball | CMJ, line drill, YYIR1 |
| Chiwaridzo et al. (20) | CS | 158 | 0 | 15\| 17 | Rugby | 20 m, 40 m, vertical jump, YYIR1, wall sit, 2 kg medicine ball throw |
| Ciacci et al. (21) | CS | 39 | 0 | 15\| 16\| 17\| 18 | Professional academy, Basketball | SJ, CMJ |
| Cobley et al. (22) | ML, 3 y | 243 | 0 | 14\| 15\| 16 | Professional academy, Rugby | 10 m, 20 m, 30 m, 60 m, CMJ, Agility 505, 20-m multistage, 2 kg medicine ball throw |
| Condello et al. (23) | CS | 108 | 0 | U13\| U15\| U17\| U19 | Professional academy, Rugby | 15 m, 2 x 15 m CoD |
|  |  |  |  |  |  |  |
| **Study** | **Design, Duration** | **B  (n)** | **G (n)** | **Age (yrs)** | **Competitive level, sport** | **Tests used** |
| Cordingley et al. (24) | L, 3 y | 306 | 0 | 13\| 14\| 15 | Ice-hockey | Broad jump, 5-10-5 shuttle run, beep test, anaerobic fitness absolute peak, handgrip |
| Coutinho et al. (25) | CS | 30 | 0 | 14\| 16 | Regional level, Football | 30 m, CMJ, 4x4 m CoD 100* |
| Craig et al. (26) | ML, | 512 | 0 | U12\| U13\| U14\| U15\| U17 | Elite, Football | 10 m, 20 m, 5 m, CMJ, YYIR1 |
| Cripps et al. (27) | L, 2 y | 160 | 0 | 16\| 18 | Selected, Australian football | CMJ, AFL agility, 20-m multistage |
| Cunha et al. (28) | CS | 34 | 0 | 15\| 17 | N/A, Football | ISO knee contraction |
| Degache et al. (29) | CS | 71 | 0 | 12\| 13\| 14\| 15 | Football | Isokinetic peak torque flexion & extension |
| DeLang et al. (30) | CS | 100 | 0 | 12\| 13\| 14\| 16\| 17 | Elite, Football | ISO adduction |
| Deprez et al. (31) | ML, 3 y | 748 | 0 | U12\| U13\| U14\| U15\| U16\| U17\| U18\| U19 | Professional academy, Football | YYIR1 |
| Deprez et al. (32) | ML, | 324 | 0 | 11\| 12\| 13\| 14 | Elite (club), Football | YYIR1 |
| Deprez et al. (33) | ML, 1-7 y | 193 | 0 | 12\| 13\| 14 | Professional academy, Football | CMJ, broad jump |
|  |  |  |  |  |  |  |
| Deprez et al. (34) | ML, 7 y | 243 | 0 | 12\| 16\| 17 | Professional academy, Football | CMJ, broad jump |
| Deprez et al. (35) | CS | 1230 | 0 | 12\| 14\| 16\| 18 | Elite, Football | 30 m, 5 m, CMJ, broad jump, T-test, YYIR1 |
| Deprez et al. (36) | ML, 5 y | 587 | 0 | 12\| 13\| 14\| 15\| 16 | Contract, Football | 30 m, 5 m, CMJ, broad jump, T-test, YYIR1 |
| Deprez et al. (37) | L, 4 y | 126 | 0 | 13\| 14\| 15\| 16 | Professional academy, Football | YYIR1 |
| Dobbs et al. (38) | CS | 206 | 0 | Pre-PHV\| Circa PHV\| Post PHV | Elite, Cricket | CMJ, squat jump, IMPT |
| Duarte et al. (39) | ML, | 290 | 0 | 11\| 13 (12\|5)\| 13\| 14\| 16\| 17 | Football | ISO knee extenstion & flexion |
| Duarte et al. (40) | ML, 5 y | 290 | 0 | 11\| 13(12.5)\| 13 (\|4)\| 14\| 16\| 17 | Football | Isokinetic peak torque flexion & extension |
| Dugdale et al. (41) | ML, 6 y | 537 | 0 | 12\| 13\| 14\| 15\| 16\| 17 | Unsuccessful, Football | 10 m, 20 m, 5 m, CMJ, YYIR1 |
| Elferink-Gemser et al. (42) | ML, 3 y | 200 | 192 | 12\| 13\| 14\| 15\| 16\| 17\| 18\| 19 | Sub-elite, Field-Hockey | ISRT |
| Elferink-Gemser et al. (43) | L, 2 y | 96 | 99 | 14 \|15\| 16\| | Sub-elite, Field-hockey | Slalom sprint 30 m, ISRT |
| Emmonds et al. (44) | CS | 0 | 93 | 11\| 13\| 15 | Elite, Football | 10 m, 30 m, CMJ, Agility 505, YYIR1, IMPT |
| Emmonds et al. (45) | CS | 0 | 104 | 12\| 13\| 14\| 15 | Elite, Football | 10 m, 30 m, CMJ, agility 505, YYIR1, IMPT |
| Eskandarifard et al. (46) | CS, | 24 | 0 | Circa\| Early | Elite, Football | CMJ |
| Faltstrom et al. (47) | CS, | 0 | 418 | 12\| 13\| 14\| 16 | Two highest division, Football | Knee extension |
| Fernandez-Galvan et al. (48) | CS, | 62 | 0 | 11\| 14 \| 17 | Professional, Football | 30 m |
| Firolli et al. (49) | CS | 186 | 0 | 12\| 13\| 15\| 18 | Football | Illinois agility |
| Forbes et al. (50) | CS | 256 | 0 | 12\| 13\| 14\| 15\| 16\| 17 | Centres of excellence, Football | Isokinetic peak torque flexion & extension |
| **Study** | **Design, Duration** | **B  (n)** | **G (n)** | **Age (yrs)** | **Competitive level, sport** | **Tests used** |
| Francini et al. (51) | CS | 68 | 0 | U14\| U15\| U16\| U17 | Semi-professional, Football | YYIR1 |
| Gabbett et al. (52) | CS | 90 | 0 | 12\| 13\| 14\| 15\| | Division 4, Rugby | 10 m, 20 m, 40 m |
| Gastin et al. (53) | CS | 96 | 0 | 12\| 14\| 15\| 18 | Australian football | 20 m, 20-m multistage |
| Gaudion et al. (54) | CS | 77 | 0 | U16\| U18 | Talent identified , Australian football | 20 m, CMJ, AFL agility test, 20-multistage, RSA total |
| Giovanni et al. | CS | 186 | 0 | 12\| 13\| 15\| 18 | Football | Illinois agility |
| Giminiani et al. (55) | L, 2 y | 57 | 0 | 13\| 14\| 15 | Giovanissimi National Championship, Football | 30 m, 15 m, SJ, CMJ, 20-m multistage |
| Gonaus et al. (56) | ML, | 4733 | 0 | 14\| 15\| 16\| 17 | Non-drafted, Football | 10 m, 20 m, 5 m, CMJ, drop jump, 5x10 shuttle run, 20-m multistage, 2 kg medicine ball throw |
| Guimaraes et al. (57) | CS | 197 | 0 | 12\| 13\| 14\| 15 | N/A, Basketball | 20 m, 5 m, CMJ, squat jump, t-test, YYIR1, handgrip |
| Guimaraes et al. (58) | ML, 2 years | 601 | 0 | 12\| 13\| 14\| 15\| 16\| 17 | Basketball | CMJ, YYRI1, handgrip |
| Hamilton et al. (59) | CS | 382 | 0 | 12\| 13\| 14\| 15\| 16\| 17\| 18 | Schoolboys, Rugby | Handgrip |
| Hammami et al. (60) | CS | 130 | 0 | 12\| 15\| 16 | Football | CMJ, broad jump, back extension |
| Hammami et al. (61) | CS | 56 | 0 | 13\| 15 | Elite, Handball | 10 m, 20 m, 30 m, 40 m, CMJ, broad jump, T-half test |
| Hansen et al. (62) | ML, 2 y | 179 | 0 | 12\| 13 | Non-elite, Football | Broad jump, leg extension, handgrip |
| Hansen et al. (63) | L, 3,5 y | 196 | 0 | 12\| 13\| 15\| 16 | Non-elite , Football | VO2max, |
| Haycraft et al. (64) | CS | 287 | 0 | 12\| 14\| 16\| 18 | National, Australian football | 10 m, 20 m, 5 m, CMJ, AFL agility, 20-m multistage |
| Hirose et al. (65) | L, 2 y | 96 | 0 | 13\| 15 | Professional academy, Football | 40 m, 5 step bounding (m), 5 x 10 m shuttle run |
| Hirose et al. (66) | CS | 0 | 103 | 12\| 14\| 15\| 16\| 18\| 19 | Football | 10 m, 5 step bounding, 5 x 10 m shuttle run |
| Holm et al. (67) | L, 5 y | 48 | 0 | 12\| 13\| 14\| 17 | N/A, Football | Isokinetic peak torque flexion & extension |
| Höner et al. (68) | CS | 13869 | 0 | U12\| U13\| U14\| U15 | Non selected, Football | 20 m, slalom agility |
| Hoshikawa et al. (69) | CS | 49 | 0 | U14\| U20 | N/A, Football | Isokinetic hip flexion |
| Huijgen et al. (70) | ML, 7 y | 498 | 0 | 12\| 13\| 14\| 15\| 16\| 17\| 18\| 19 | Football | Shuttle sprint 30 m 3 x 180* |
| Ishøi et al. (71) | CS | 95 | 0 | 13\| 14\| 15\| 16\| 18 | Elite, Football | Hamstring & quadriceps strength with dynamometer |
| Jones et al. (72) | CS | 108 | 0 | 12\| 13\| 14\| 15\| 16\| 17 | Elite, Football | Isometric adduction and abduction, knee flexion |
| Jorge et al. (73) | CS | 43 | 0 | 16\| 18 | Football | Bangsbo modified sprint test, RSA, fatigue index, percentage decrement |
| Keiner et al. (74) | L, 2 y | 105 | 0 | 12\| 13\| 14 | Professional academy, Football | SJ, CMJ |
| Keiner et al. (75) | L, 2 y | 112 | 0 | 15\| 17\| 19 | Professional academy, Football | Back squat, front squat |
| **Study** | **Design, Duration** | **B  (n)** | **G (n)** | **Age (yrs)** | **Competitive level, sport** | **Tests used** |
| Keiner et al. (76) | CS | 141 | 0 | 11\| 12\| 13\| 14\| 15\| 16\| 17\| 18\| 19 | Professional academy, Football | Back squat, front squat |
| Keiner et al. (77) | CS | 72 | 0 | 17\| 18 | Elite, Football | CMJ, squat jump |
| Keiner et al. (78) | L, 2 y | 114 | 0 | U14\| U15\| U16\| U17\| U18\| U19\| U20 | Elite, Football | Back squat |
| Kelly et al. (79) | CS | 53 | 0 | 16\| 17\| 20 | Australian football | CMJ, SL bound, 3k time trial, YYIR2, back squat, RDL, bench press |
| Knoop et al. (80) | CS | 34 | 0 | U14\| U19 | Football | 10 m, CMJ |
| Kobal et al. (81) | CS | 46 | 0 | 14\| 17\| 19 | High level club, Rugby | 10 m, 20 m, 40 m, CMJ, squat jump, pro agility, YYER1 |
| Kokinda et al. (82) | CS | 46 | 0 | 16\| 19 | Ice-hockey | Wingate |
| Konieczna et al. (83) | L, 3 y | 0 | 0 | 13\| 14\| 15 | Football | PWC170 test, cooper test |
| Koryahin et al. (84) | CS | 767 | 0 | 11\| 12\| 13\| 14\| 15\| 16\| 17\| 18\| 19 | Masters of sport, Basketball | 20 m, 6 m, broad jump, running on court 3 x 40 s |
| Kos et al. (85) | CS | 45 | 0 | 12\| 14\| 16 | State lvl, Basketball | Line drill, YYIR1 |
| Kruse et al. (86) | CS | 485 | 0 | U12\| U13\| U14\| U15 | Rugby | 10 m, 30 m, 40 m, CMJ |
| Landgraff et al. (87) | L, 4 y | 87 | 27 | 12\| 13\| 15 | Football | VO2max, time to exhaustion |
| Leao et al. (88) | CS, | 65 | 0 | U16\| U17\| U19 | Football | CMJ, YYIR2 |
| Lehnert et al. (89) | CS | 45 | 0 | U16\| U17\| U18 | Professional academy, Football | Isokinetic peak torque flexion & extension |
| Leiter et al. (90) | CS | 197 | 0 | 13\| 14\| 15\| 16\| 17 | Ice-hockey | Wingate |
| Lemos et al. (91) | CS | 33 | 0 | 18\| 20 | Beach handball | 15 m, broad jump, handgrip |
| Leppanen et al. (92) | CS, | 207 | 46 | 12\| 13 | Football | 30 m, CMJ, YYIR1 |
| Letter et al. (93) | L, 7 years | 309 | 174 | 18\| 19\| 20 | National level, Cricket | CMJ, IMPT |
| Leyhr et al. (94) | L, 4 y | 1134 | 0 | U12\| U13\| U14\| U15 | Non elite, Football | 20 m, agility slalom |
| Leyhr et al. (95) | CS | 0 | 1832 | U12\| U13\| U14\| U15 | Non-professional, Football | Slalom agility |
| Lima et al. (96) | CS | 20 | 0 | 12\| 14 | Elite, Basketball | CMJ, YYIR1 |
| Lolli et al. (97) | CS, | 62 | 0 | 12\| 13\| 14\| 15\| 16\| 17\| 18 | academy, Football | 10 m, CMJ |
| Loturco et al. (98) | CS | 133 | 0 | 15\| 16\| 19 | Elite, Football | 5 m, squat jump, CMJ, 4x5 m CoD 45* |
| Łuszczyk et al. (99) | L, 2 y | 36 | 0 | 16\| 17\| 18 | N/A, Handball | Wingate |
| Markovic et al. (100) | CS | 117 | 0 | U13\| U14\| U15\| U16\| U17\| U18\| U19 | First league croatia, Football | YYIR1 |
| Markovic et al.(101) | CS | 123 | 0 | U12\| U13\| U14\| U15\| U16\| U17\| U18 | Elite, Football | 20 m, hamstring eccentric strength |
| Martins et al. (102) | CS | 62 | 0 | 13\| 15\| 17 | Elite, Football | 10 m, 30 m, broad jump, carminatti's test, RSA |
| Matthys et al. (103) | L, 3 y | 282 | 0 | 13\| 14\| 15\| 16\| 17 | Non-elite, Handball | 10 m, 20 m, 30 m, 5 m, CMJ arms, 5 jump test, 10 x 5 shuttle run, YYIR1, handgrip |
| Matthys et al. (104) | CS | 471 | 0 | 14\| 15\| 16 | Handball | 10 m, 20 m, CMJ arms, 5 jump test, 10x5 shuttle run |
| **Study** | **Design, Duration** | **B  (n)** | **G (n)** | **Age (yrs)** | **Competitive level, sport** | **Tests used** |
| Mendez-Villanueva et al. (105) | CS | 61 | 0 | 12\| 14\| 17 | Elite, Football | 40 m max speed, UMTT |
| Mendez-Villanueva et al. (106) | CS | 61 | 0 | 13\| 15\| 17 | Elite, Football | 10 m, 20 m flying, RSA |
| Metaxas et al. (107) | CS | 19 | 0 | 13\| 15 | Elite, Football | Time to exhaustion, VO2max, isokinetic peak torque extension & flexion |
| Metaxas et al. (108) | CS | 17 | 0 | 13\| 14 | N/A, Football | 10 m, bangsbo modified sprint test, |
| Mirkov et al. (109) | L, 3 y | 293 | 0 | 12\| 13\| 14 | Football | CMJ, broad jump, 10x5 shuttle run |
| Morris et al. (110) | CS | 0 | 0 | 12\| 14\| 16 | Elite, Football | CMJ, arrowhead agility, IMPT |
| Nedeljkoic et al. (111) | CS | 478 | 0 | 12\| 13\| 14\| 15\| 16\| 17 | Professional academy, Football | CMJ, broad jump, 10x5 shuttle run, knee extension, hip flexion |
| Nguyen et al. (112) | L, 2 y | 0 | 42 | 14\| 15\| 16 | Football | Hip abduction, hip extension |
| Niederer et al. (113) | CS | 129 | 0 | U12\| U13\| U14\| U15\| U16\| U17\| U19 | Elite, Football | 10 m, 30 m, CMJ, Zig-Zag 4x5 m 100*, isometric knee extension & flexion |
| Nikolaidis et al. (114) | CS | 275 | 0 | 13\| 14\| 15\| 17\| 18\| 20\| 21 | Competitive club, Football | PWC170 test |
| Nikolaidis et al. (115) | CS | 58 | 0 | 14\| 16 | Top national, Handball | CMJ, squat jump, wingate peak power |
| Noon et al. (116) | CS | 348 | 0 | 12\| 13\| 14\| 15\| 16 | Football | 30 m, 15 m, CMJ, arrowhead agility |
| Nutt et al. (117) | CS, | 249 | 109 | 12\| 13\| 14\| 15\| U19 | League and/or union, Football | handgrip |
| Nutton et al. (118) | CS | 382 | 0 | 12\| 13\| 14\| 15\| 16\| 17\| 18 | School rugby, Rugby | Handgrip |
| Parpa et al. (119) | CS, | 110 | 0 | 12\| 13\| 14 | UEFA-licensed coaches, Football | 10 m, 30 m, handgrip |
| Peek et al. (120) | CS | 47 | 0 | 12\| 13\| 14\| 15 | Football | Isometric knee extension & knee flexion |
| Perroni et al. (121) | CS | 0 | 0 | 14\| 16\| 18 | Football | RSA |
| Petridis et al. (122) | CS | 365 | 0 | 16\| 17\| 18 | National competitions, Football | Squat jump, CMJ |
| Pizzigalli et al. (123) | CS | 90 | 0 | U14\| U15\| U16\| U17\| U18\| U20 | Elite, Basketball | Handgrip |
| Quatman et al. (124) | L, 2 y | 34 | 32 | 13\| 14\| 15 | N/A, Basketball | Drop vertical jump |
| Read et al. (125) | CS | 747 | 0 | 11\| 12\| 13\| 14\| 15\| 16\| 18 | Elite, Soccer | Horizontal jump SL |
| Read et al. (126) | CS | 347 | 0 | 12\| 14\| 16 | Elite, Football | SL jump |
| Rebelo-Goncalves et al. (127) | ML, | 71 | 0 | 12\| 14\| 16\| 18 | Elite, Football | 20-m multistage |
| Reinikainen et al. (128) | ML, 2 y | 212 | 0 | 13\| 14\| 15\| 16\| 17 | Australian football | 20 m, 40 m, CMJ arms, AFL agility, 2 k time trial |
| Ritsche et al. (129) | CS | 85 | 0 | 13\| 14 | Regional to national, Football | 10 m, 30 m |
| Roe et al. (130) | CS | 271 | 0 | U13\| U14\| U15\| U16\| Minor | Sub elite, Gaelic football | YYIR1 |
| Roe et al. (131) | CS | 193 | 0 | 14\| 15\| 16\| 17\| 20 | Elite, Gaelic football | Hamstring eccentric strength |
| Roescher et al. (132) | ML, | 229 | 0 | 14\| 15\| 16\| 17\| 18 | Non-professional, Football | ISRT |
| **Study** | **Design, Duration** | **B  (n)** | **G (n)** | **Age (yrs)** | **Competitive level, sport** | **Tests used** |
| Salinero et al. (133) | CS | 114 | 0 | 14\| 16\| 19 | Elite, Football | CMJ, RSA |
| Sander et al. (134) | L, 2 y | 272 | 0 | 13\| 15\| 17\| 19 | Professional academy, Football | 10 m, 20 m, 30 m, 15 m, front squat, back squat |
| Saward et al. (135) | ML, 11 y | 5970 | 0 | 12\| 13\| 14\| 15\| 16\| 17\| 18\| 19 | Elite, Football | 20 m max speed, CMJ arms, 20.8-m slaslom agility, 20-m multistage |
| Sekine et al. (136) | L, 3 y | 130 | 0 | 13\| 14 | N/A, Basketball | CMJ arms |
| Silva et al. (137) | CS, | 123 | 0 | 14\| 15\| 16\| 17\| 18 | Professional, Football | 10 m, 30 m, CMJ |
| Śliwowski et al. (138) | L, 1 year | 66 | 0 | 17\| 18\| 15\| 16 | Football | Threshold running speed (4 mmol) |
| Söderman et al. (139) | CS | 0 | 51 | 15\| 17 | Elite, Football | Isokinetic peak torque flexion & extension |
| Spencer et al. (140) | CS | 119 | 0 | U11\| U12\| U13\| U14\| U15\| U16\| U17\| U18 | Professional academy, Football | 15 m max speed, CMJ, CMJ arms, 15 m agility run (m/s), RSA total, 20-m multistage |
| Stojomenovic et al. (141) | L, 3 y | 0 | 25 | 14 (13\|8)\| 15\|16 | Training group, Basketball | VO_2_**_-_**max |
| Sugimoto et al. (142) | CS | 0 | 115 | <12 y\| 13-16 y\| >17 y | N/A, Football | Hamstring & quadriceps strength with dynamometer |
| Saavedra et al. (143) | CS | 0 | 57 | 14\| 16\| 18 | Elite, Handball | 10 m, 30 m, CMJ, YYIR2, handgrip |
| Teixeira et al. (144) | CS | 92 | 0 | U12\| U14\| U16 | Professional academy, Football | Carminattis's test |
| Te Wierike et al. (145) | ML, | 130 | 0 | 14\| 15\| 16\| 17\| 18\| 19 | Basketball | Vertical jump (yardstick), shuttle run 30 m 3x180*, ISRT |
| Till et al. (146) | L, 3 y | 243 | 0 | 14\| 15\| 16 | Rugby | 10 m, 20 m, 30 m, 60 m, CMJ, Agility 505, 20-m multistage, 2 kg medicine ball throw |
| Till et al. (147) | L, 3 y | 9 | 0 | 13\| 14\| 15 \| 16 | Rugby | 10 m, 20 m, 30 m, 60 m, CMJ, Agility 505, 20-m multistage, 2 kg medicine ball throw |
| Till et al. (148) | L, 3 y | 243 | 0 | 13\| 14\| 15 | Regional representative level, Rugby | 10 m, 20 m, 30 m, 60 m, CMJ, Agility 505, 20-m multistage, 2 kg medicine ball throw |
| Till et al. (149) | ML, | 257 | 0 | U16\| U17\| U18\| U19\| U20 | Professional academy, Rugby | 10 m, 20 m, CMJ, YYIR1, back squat, bench press |
| Till et al. (150) | ML, 3 y | 195 | 0 | U14\| U16\| U18\| U20 | Professional academy, Rugby | 10 m, 20 m, CMJ, YYIR1 |
| Till et al. (151) | L, 5 y | 75 | 0 | U17\| U18\| U19 | Professional, Rugby | 10 m, 20 m, CMJ, YYIR1, back squat, bench press |
| Till et al. (152) | L, 4 y | 318 | 0 | 16\| 17\| 18\| 19\| 20 | 4 consecutive years, Rugby | 10 m, 20 m, CMJ, YYIR1, back squat, bench press |
| Till et al. (153) | ML, 5 y | 206 | 0 | 12\| 13\| 14\| 15\| 16 | Rugby | 10 m, 20 m, CMJ, 20-m multistage, 2 kg medicine ball throw |
| Till et al. (154) | CS | 580 | 0 | 13\| 14\| 15 | Professional, Rugby | 10 m, 20 m, 30 m, 60 m, CMJ, Agility 505, 20-m multistage, 2 kg medicine ball throw |
| Till et al. (155) | L, 3 y | 153 | 0 | 14\| 15\| 16 | Professional, Rugby | 10 m, 20 m, 30 m, 60 m, CMJ, Agility 505, 20-m multistage, 2 kg medicine ball throw |
| Toong et al. (156) | CS | 323 | 367 | 10\| 11\| 12\| 13\| 14\| 15\| 16 | Ice-hockey | Handgrip |
| **Study** | **Design, Duration** | **B  (n)** | **G (n)** | **Age (yrs)** | **Competitive level, sport** | **Tests used** |
| Tribolet et al. (157) | CS | 266 | 0 | 13\| 14\| 15 | Elite, Australian football | Broad jump, T-test, knee push ups |
| Vaeyens et al. (158) | ML, 5 y | 374 | 0 | U13\| U14\| U15\| U16 | Non-elite, Football | 30 m, VJ, broad jump, shuttle run, 20-m multistage, shuttle tempo run, bent arm hang |
| Valente-dos-Santos et al. (159) | ML, | 366 | 0 | 12\| 13\| 14\| 15\| 16\| 17\| 18 | N/A, Football | CMJ, RSA, 20-m multistage |
| Valente-dos-Santos et al. (160) | ML, | 332 | 0 | 12\| 13\| 14\| 15\| 16\| 17\| 18 | N/A, Football | CMJ, 10x5 shuttle run, 20-m multistage |
| Valente-dos-Santos et al. (161) | ML, 3-5 y | 366 | 0 | 12\| 13\| 14\| 15\| 16\| 17\| 18 | Football | 20-m multistage |
| Valente-dos-Santos et al. (162) | ML, 3-5 y | 366 | 0 | 12\| 14\| 16\| 18 | National level, Football | 10 x 5 m shuttle run |
| Vandendriessche et al. (163) | CS | 73 | 0 | 15\| 16 | Futures, Football | 10 m, 20 m, 30 m, 5 m, CMJ, broad jump, T-test |
| Vargas et al. (164) | CS | 0 | 66 | 12\| 14\| 16\| 18 | Regional and national, Football | Isokinetic peak torque flexion & extension |
| Vera Assaoka et al. (165) | CS | 76 | 0 | 12\|15 | N/A, Football | CMJ, 5 multiple bounds, illinois agility, 2,4 k time trial, 5RM squat |
| Vernillo et al. (166) | CS | 88 | 0 | U14\| U15\| U17 | Sub-elite, Basketball | YYIR1 |
| Visnapuu et al. (167) | CS | 99 | 0 | 12-13 y\| 14-15 y\| 16-17 y | National championship, Handball | 30 m, CMJ, CMJ arms, 4x10 m shuttle run, 1 kg medicine ball throw |
| Waldron et al. (168) | ML, 3 y | 57 | 0 | 15\| 16\| 17 | Elite, Rugby | 30 max speed, CMJ, predicted VO2max, |
| Waldron et al. (169) | L, 3 y | 39 | 0 | 15\| 16\| 17 | Elite, Rugby | 20 m, CMJ, 20-m multistage |
| Williams et al. (170) | ML, 3 y | 200 | 0 | U12\| U13\| U14\| U15\| U16 | Centre of excellence, Football | 10 m, 30 m CMJ arms |
| Wollin et al. (171) | L, 22 months | 27 | 0 | 15\| 16 | Football | Hip adduction, hip abduction |
| Woodcock et al. (172) | ML, | 2201 | 0 | 12\| 13\| 14\| 15\| 16\| 17\| 18\| 19\| 20 | Elite, Football | CMJ, broad jump |
| Woods et al. (173) | CS | 134 | 0 | 15\| 17 | Non-talent identified, Australian football | 20 m, CMJ 20-m multistage |
| Wright et al. (174) | L, 3 y | 0 | 42 | 12\| 13\| 14 | Elite, Football | 20 m, 5 m, CoD speed, RSA |
| Yang et al. (175) | CS, | 334 | 0 | 13\| 14\| 15 | Elite, Football | 10 m, 30 m, broad jump, YYIR1 |

# **Reference list**

1. Agrebi B, Tkatchuk V, Hlila N, Mouelhi E, Belhani A. Impact of specific training and competition on myocardial structure and function in different age ranges of male handball players. PLoS One. 2015 Dec 1;10(12).

2. Alvurdu S, Baykal C, Akyildiz Z, Şenel Ö, Silva AF, Conte D, et al. Impact of Prolonged Absence of Organized Training on Body Composition, Neuromuscular Performance, and Aerobic Capacity: A Study in Youth Male Soccer Players Exposed to COVID-19 Lockdown. Int J Environ Res Public Health. 2022;19(3):1148.

3. Andrade MS, Junqueira MS, Andre Barbosa De Lira C, Vancini RL, Seffrin A, Nikolaidis PT, et al. Age-related differences in torque in angle-specific and peak torque hamstring to quadriceps ratios in female soccer players from 11 to 18 years old: Α Cross-sectional study. Res Sports Med. 2021;29(1):77–89.

4. Andrade MS, de Lira CAB, Vancini RL, de Almeida AA, Benedito-Silva AA, da Silva AC. Profiling the isokinetic shoulder rotator muscle strength in 13- to 36-year-old male and female handball players. Phys Ther Sport. 2013;14(4):246–52.

5. Atan SA, Foskett A, Ali A. Motion analysis of match play in New Zealand U13 to U15 age-group soccer players. Int J Sports Physiol Perform. 2016;30(9):2416–23.

6. Barr MJ, Sheppard JM, Gabbett TJ, Newton RU. Long-term training-induced changes in sprinting speed and sprint momentum in elite rugby union players. J Strength Cond Res. 2014;28(10):2724–31.

7. Baxter-Jones A, Goldstein H, Helms P. The development of aerobic power in young athletes. J Appl Physiol (1985). 1993;75(3):1160–7.

8. Bennett H, Chalmers S, Arnold J, Milanese S, Blacket C, Niculescu A, et al. The Relationship Between Performance and Injury in Junior Australian Football Athletes. Int J Sports Physiol Perform. 2022 May 1;17(5):761–7.

9. Bidaurrazaga-Letona I, Carvalho HM, Lekue JA, Santos-Concejero J, Figueiredo AJ, Gil SM. Longitudinal field test assessment in a Basque soccer youth academy: A multilevel modeling framework to partition effects of maturation. Int J Sports Med. 2015;36(3):234–40.

10. Bona CC, Filho HT, Izquierdo M, Ferraz RMP, Marques MC. Peak torque and muscle balance in the knees of young U-15 and U-17 soccer athletes playing various tactical positions. J Sports Med Phys Fitness. 2017;57(7–8):923–9.

11. Brent JL, Myer GD, Ford KR, Paterno M V, Hewett TE. The Effect of Sex and Age on Isokinetic Hip-Abduction Torques. J Sport Rehabil. 2013;22(1):41–6.

12. Buchanan PA, Vardaxis VG. Sex-Related and Age-Related Differences in Knee Strength of Basketball Players Ages 11-17 Years. J Athl Train. 2003;38(3):231–7.

13. Buchheit M, Mendez-Villanueva A. Reliability and stability of anthropometric and performance measures in highly-trained young soccer players: effect of age and maturation. J Sports Sci. 2013 Aug;31(12):1332–43.

14. Byrne LM, Byrne PJ, Byrne EK, Byrne AP, Coyle C. Cross-Sectional Study of the Physical Fitness and Anthropometric Profiles of Adolescent Hurling, Camogie, and Gaelic Football Players. J Strength Cond Res. 2021;36(12):3422–31.

15. Cardoso de Araújo M, Baumgart C, Freiwald J, Hoppe MW. Nonlinear sprint performance differentiates professional from young soccer players. J Sports Med Phys Fitness. 20170222nd ed. 2018;58(9):1204–10.

16. Carvalho HM, Bidaurrazaga-Letona I, Lekue JA, Amado M, Figueiredo AJ, Gil SM. Physical growth and changes in intermittent endurance run performance in young male basque soccer players. Res Sports Med. 2014;22(4):408–24.

17. Carvalho HM, Gonçalves CE, Grosgeorge B, Paes RR. Validity and usefulness of the Line Drill test for adolescent basketball players: a Bayesian multilevel analysis. Research in Sports Medicine. 2017;25(3):333–44.

18. Carvalho HM, Lekue JA, Gil SM, Bidaurrazaga-Letona I. Pubertal development of body size and soccer-specific functional capacities in adolescent players. Res Sports Med. 20170817th ed. 2017;25(4):421–36.

19. Carvalho HM, Leonardi TJ, Soares ALA, Paes RR, Foster C, Gonçalves CE. Longitudinal changes of functional capacities among adolescent female basketball players. Front Physiol. 2019;10:339.

20. Chiwaridzo M, Ferguson GD, Smits-Engelsman BCM. Anthropometric, physiological characteristics and rugby-specific game skills of schoolboy players of different age categories and playing standards. BMC Sports Sci Med Rehabil. 2020;12(1):3.

21. Ciacci S, Bartolomei S. The effects of two different explosive strength training programs on vertical jump performance in basketball. J Sports Med Phys Fitness. 2018;58(10):1375–82.

22. Cobley SP, Till K, O’Hara J, Cooke C, Chapman C. Variable and Changing Trajectories in Youth Athlete Development: Further Verification in Advocating a Long-term Inclusive Tracking Approach. J Strength Cond Res. 2014;28(7):1959–70.

23. Condello G, Minganti C, Lupo C, Benvenuti C, Pacini D, Tessitore A. Evaluation of change-of-direction movements in young rugby players. Int J Sports Physiol Perform. 20120731st ed. 2013;8(1):52–6.

24. Cordingley DM, Sirant L, MacDonald PB, Leiter JR. Three-Year Longitudinal Fitness Tracking in Top-Level Competitive Youth Ice Hockey Players. J Strength Cond Res. 2019;33(11):2909–12.

25. Coutinho D, Santos S, Gonçalves B, Travassos B, Wong DP, Schöllhorn W, et al. The effects of an enrichment training program for youth football attackers. PLoS One. 20180613th ed. 2018;13(6):e0199008.

26. Craig TP, Swinton P. Anthropometric and physical performance profiling does not predict professional contracts awarded in an elite Scottish soccer academy over a 10-year period. Eur J Sport Sci. 2021;21(8):1101–10.

27. Cripps AJ, Banyard HG, Woods CT, Joyce C, Hopper LS. Does the longitudinal development of physical and anthropometric characteristics associate with professional career attainment in adolescent Australian footballers? Int J Sports Sci Coach. 2020;15(4):506–11.

28. Cunha G dos S, Vaz MA, Herzog W, Geremia JM, Leites GT, Reischak-Oliveira Á. Maturity status effects on torque and muscle architecture of young soccer players. J Sports Sci. 2020;38(11–12):1286–95.

29. Degache F, Richard R, Edouard P, Oullion R, Calmels P. The relationship between muscle strength and physiological age: A cross-sectional study in boys aged from 11 to 15. Ann Phys Rehabil Med. 2010;53(3):180–8.

30. DeLang MD, Garrison JC, Hannon JP, McGovern RP, Christoforetti J, Thorborg K. Short and long lever adductor squeeze strength values in 100 elite youth soccer players: Does age and previous groin pain matter? PhysTher Sport. 2020;46:243–8.

31. Deprez D, Vaeyens R, Coutts AJ, Lenoir M, Philippaerts R. Relative age effect and Yo-Yo IR1 in youth soccer. Int J Sports Med. 2012;33(12):987–93.

32. Deprez D, Valente-dos-Santos J, Coelho e Silva M, Lenoir M, Philippaerts RM, Vaeyens R. Modeling developmental changes in the Yo-Yo Intermittent Recovery Test Level 1 in elite pubertal soccer players. Int J Sports Physiol Perform. 2014;9(6):1006–12.

33. Deprez D, Valente-Dos-Santos J, Coelho-E-Silva MJ, Lenoir M, Philippaerts R, Vaeyens R. Multilevel development models of explosive leg power in high-level soccer players. Med Sci Sports Exerc. 2015;47(7):1408–15.

34. Deprez D, Valente-Dos-Santos J, Coelho-E-Silva MJ, Lenoir M, Philippaerts R, Vaeyens R. Longitudinal Development of Explosive Leg Power from Childhood to Adulthood in Soccer Players. Int J Sports Med. 2015;36(8):672–9.

35. Deprez D, Fransen J, Boone J, Lenoir M, Philippaerts R, Vaeyens R. Characteristics of high-level youth soccer players: variation by playing position. J Sports Sci. 2015;33(3):243–54.

36. Deprez D, Fransen J, Lenoir M, Philippaerts R, Vaeyens R. A retrospective study on anthropometrical, physical fitness, and motor coordination characteristics that influence dropout, contract status, and first-team playing time in high-level soccer players aged eight to eighteen years. J Strength Cond Res. 2015;29(6):1692–704.

37. Deprez D, Buchheit M, Fransen J, Pion J, Lenoir M, Philippaerts RM, et al. A Longitudinal Study Investigating the Stability of Anthropometry and Soccer-Specific Endurance in Pubertal High-Level Youth Soccer Players [Internet]. Vol. 14, ©Journal of Sports Science and Medicine. 2015. Available from: http://www.jssm.org

38. Dobbs IJ, Oliver JL, Wong MA, Moore IS, Lloyd RS. Movement competency and measures of isometric and dynamic strength and power in boys of different maturity status. Scand J Med Sci Sports. 2020 Nov 1;30(11):2143–53.

39. Duarte JP, Valente-Dos-Santos J, Coelho-E-Silva MJ, Malina RM, Deprez D, Philippaerts R, et al. Developmental Changes in Isometric Strength: Longitudinal Study in Adolescent Soccer Players. Int J Sports Med. 2018;39(9):688–95.

40. Duarte JP, Valente-dos-Santos J, Costa D, Coelho-e-Silva MJ, Deprez D, Philippaerts R, et al. Multilevel modelling of longitudinal changes in isokinetic knee extensor and flexor strength in adolescent soccer players. Ann Hum Biol. 2018;45(5):453–6.

41. Dugdale JH, Sanders D, Myers T, Williams AM, Hunter AM. Progression from youth to professional soccer: A longitudinal study of successful and unsuccessful academy graduates. Scand J Med Sci Sports. 2021;31(S1):73–84.

42. Elferink-Gemser MT, Visscher C, Van Duijn MAJ, Lemmink KAPM. Development of the interval endurance capacity in elite and sub-elite youth field hockey players. Br J Sports Med. 2006;40(4):340–5.

43. Elferink-Gemser MT, Visscher C, Lemmink KAPM, Mulder T. Multidimensional performance characteristics and standard of performance in talented youth field hockey players: A longitudinal study. J Sports Sci. 2007;25(4):481–9.

44. Emmonds S, Sawczuk T, Scantlebury S, Till K, Jones B. Seasonal Changes in the Physical Performance of Elite Youth Female Soccer Players. J Strength Cond Res. 2020;34(9):2636–43.

45. Emmonds S, Scantlebury S, Murray E, Turner L, Robsinon C, Jones B. Physical Characteristics of Elite Youth Female Soccer Players Characterized by Maturity Status. J Strength Cond Res. 2020;34(8):2321–8.

46. Eskandarifard E, Silva R, Nobari H, Clemente FM, Pérez-Gómez J, Figueiredo AJ. Maturational effect on physical capacities and anabolic hormones in under-16 elite footballers: a cross-sectional study. Sport Sci Health. 2022;18(2):297–305.

47. Fältström A, Skillgate E, Tranaeus U, Weiss N, Källberg H, Lyberg V, et al. Normative values and changes in range of motion, strength, and functional performance over 1 year in adolescent female football players: Data from 418 players in the Karolinska football Injury Cohort study. Phys Ther Sport. 2022;58:106–16.

48. Fernández-Galván LM, Jiménez-Reyes P, Cuadrado-Peñafiel V, Casado A. Sprint Performance and Mechanical Force-Velocity Profile among Different Maturational Stages in Young Soccer Players. Int J Environ Res Public Health. 2022 Feb 1;19(3):1412.

49. Firolli G, Mitrotasios M, Iuliano E, Pistone EM, Aquino G, Calcagno G, et al. Agility and change of direction in soccer: Differences according to the player ages. J Sports Med Phys Fitness. 2017;57(12):1597–604.

50. Forbes H, Sutcliffe S, Lovell A, McNaughton LR, Siegler JC. Isokinetic thigh muscle ratios in youth football: effect of age and dominance. Int J Sports Med. 20090319th ed. 2009;30(8):602–6.

51. Francini L, Rampinini E, Bosio A, Connolly D, Carlomagno D, Castagna C. Association Between Match Activity, Endurance Levels and Maturity in Youth Football Players. Int J Sports Med. 20190704th ed. 2019;40(9):576–84.

52. Gabbett TJ. Use of Relative Speed Zones Increases the High-Speed Running Performed in Team Sport Match Play. J Strength Cond Res. 2015;29(12):3353–9.

53. Gastin PB, Bennett G, Cook J. Biological maturity influences running performance in junior Australian football. J Sci Med Sport. 2013;16(2):140–5.

54. Gaudion SL, Doma K, Sinclair W, Banyard HG, Woods CT. Identifying the Physical Fitness, Anthropometric and Athletic Movement Qualities Discriminant of Developmental Level in Elite Junior Australian Football: Implications for the Development of Talent. J Strength Cond Res. 2017;31(7):1830–9.

55. Di Giminiani R, Visca C. Explosive strength and endurance adaptations in young elite soccer players during two soccer seasons. PLoS One. 20170213th ed. 2017;12(2):e0171734.

56. Gonaus C, Müller E. Using physiological data to predict future career progression in 14- to 17-year-old Austrian soccer academy players. J Sports Sci. 2012;30(15):1673–82.

57. Guimarães E, Baxter-Jones ADG, Williams AM, Tavares F, Janeira MA, Maia J. The role of growth, maturation and sporting environment on the development of performance and technical and tactical skills in youth basketball players: The INEX study. J Sports Sci. 2021;39(9):979–91.

58. Guimarães E, Baxter-Jones ADG, Williams AM, Tavares F, Janeira MA, Maia J. The effects of body size and training environment on the physical performance of adolescent basketball players: the INEX study. Ann Hum Biol. 2023;50(1):26–34.

59. Hamilton DF, Gatherer D, Jenkins PJ, Maclean JG, Hutchison JD, Nutton RW, et al. Age-related differences in the neck strength of adolescent rugby players: A cross-sectional cohort study of Scottish schoolchildren. Bone Joint Res. 20120701st ed. 2012;1(7):152–7.

60. Hammami R, Chaouachi A, Makhlouf I, Granacher U, Behm DG. Associations between balance and muscle strength, power performance in male youth athletes of different maturity status. Pediatr Exerc Sci. 2016;28(4):521–34.

61. Hammami R, Sekulic D, Selmi MA, Fadhloun M, Spasic M, Uljevic O, et al. Maturity status as a determinant of the relationships between conditioning qualities and preplanned agility in young handball athletes. J Strength Cond Res. 2018;32(8):2302–13.

62. Hansen L, Bangsbo J, Twisk J, Klausen AK, Klausen K. Development of muscle strength in relation to training level and testosterone in young male soccer players. J Appl Physiol. 1999;87(3):1141–7.

63. Hansen L, Klausen K. Development of aerobic power in pubescent male soccer players related to hematocrit, hemoglobin and maturation. A longitudinal study. J Sports Med Phys Fitness. 2004;44(3):219–23.

64. Haycraft JAZ, Kovalchik S, Pyne DB, Robertson S. Relationships between physical testing and match activity profiles across the Australian Football League participation pathway. Int J Sports Physiol Perform. 2019;14(6):771–8.

65. Hirose N, Seki T. Two-year changes in anthropometric and motor ability values as talent identification indexes in youth soccer players. J Sci Med Sport. 2016;19(2):158–62.

66. Hirose N, Nakahori C. Age differences in change-of-direction performance and its subelements in female football players. Int J Sports Physiol Perform. 2015;10(4):440–5.

67. Holm I, Steen H, Olstad M. Isokinetic muscle performance in growing boys from pre-teen to maturity. An eleven-year longitudinal study. Isokinet Exerc Sci. 2005;13(2):153–8.

68. Höner O, Murr D, Larkin P, Schreiner R, Leyhr D. Nationwide Subjective and Objective Assessments of Potential Talent Predictors in Elite Youth Soccer: An Investigation of Prognostic Validity in a Prospective Study. Front Sports Act Living. 2021;3:638227.

69. Hoshikawa Y, Iida T, Ii N, Muramatsu M, Nakajima Y, Chumank K, et al. Cross-sectional area of psoas major muscle and hip flexion strength in youth soccer players. Eur J Appl Physiol. 2012;112(10):3487–94.

70. Huijgen BCH, Elferink-Gemser MT, Post W, Visscher C. Development of dribbling in talented youth soccer players aged 12-19 years: A longitudinal study. J Sports Sci. 2010;28(7):689–98.

71. Ishøi L, Krommes K, Nielsen MF, Thornton KB, Hölmich P, Aagaard P, et al. Hamstring and quadriceps muscle strength in youth to senior elite soccer: A cross-sectional study including 125 players. Int J Sports Physiol Perform. 2021;16(10):1538–44.

72. Jones S, Clair Z, Wrigley R, Mullen R, Andersen TE, Williams M. Strength development and non-contact lower limb injury in academy footballers across age groups. Scand J Med Sci Sports. 2021 Mar 1;31(3):679–90.

73. Jorge G, Garrafoli MT, Cal Abad CC. Seasonal Repeated Sprint Ability With Change of Direction Variations in U17 and U20 Elite Brazilian Soccer Players: A Comparative Study. J Strength Cond Res. 2020;34(5):1431–9.

74. Keiner M, Sander A, Wirth K, Schmidtbleicher D. The impact of 2 years of additional athletic training on the jump performance of young athletes. Sci Sports. 2014;29(4):e39–46.

75. Keiner M, Sander A, Wirth K, Schmidtbleicher D. Long-term strength training effects on change-of-direction sprint performance. J Strength Cond Res. 2014;28(1):223–31.

76. Keiner M, Sander A, Wirth K, Caruso O, Immesberger P, Zawieja M. Strength performance in youth: trainability of adolescents and children in the back and front squats. J Strength Cond Res. 2013;27(2):357–62.

77. Keiner M, Sander A, Wirth K, Hartmann H. Differences in the performance tests of the fast and slow stretch and shortening cycle among professional, amateur and elite youth soccer players. J Hum Sport Exerc. 2015;10(2):563–70.

78. Keiner M, Sander A, Bobsled G, Association L, Hartmann H, Mickel C. Do long-term strength training and age affect the performance of drop jump in adolescents? Separating the effects of training and age. J Austr Strength Cond. 2018;26(4):24–38.

79. Kelly VG, Leveritt MD, Brennan CT, Slater GJ, Jenkins DG. Prevalence, knowledge and attitudes relating to β-alanine use among professional footballers. J Sci Med Sport. 20160623rd ed. 2017;20(1):12–6.

80. Knoop M, Fernandez-Fernandez J, Ferrauti A. Evaluation of a specific reaction and action speed test for the soccer goalkeeper. J Strength Cond Res. 2013;27(8):2141–8.

81. Kobal R, Nakamura FY, Moraes JE, Coelho M, Kitamura K, Cal Abad CC, et al. Physical performance of brazilian rugby players from different age categories and competitive levels. J Strength Cond Res. 2016;30(9):2433–9.

82. Kokinda M, Kandráč R, Čech P. Analysis of age-related changes of anaerobic power in ice hockey. J Phys Educ Sport. 2020;20(6):3546–52.

83. Konieczna A, Radzimiński Ł, Paszulewicz J, Lopez-Sanchez GF, Dragos P, Jastrzębski Z. Physical capacity and body composition in 13-16 year old soccer players during three-year training cycle. Balt J Health Phys Act. 2019;11(4):47–57.

84. Koryahin V, Iedynak G, Blavt O, Galamandjuk L, Ludovyk T, Stadnyk V, et al. The main aspects of the implementation of technical and physical training of basketball players. J Phys Educ Sport. 2019;19:358–62.

85. Kos LD, Soares AL, Mendes FG, Lima AB, Collet C, Nascimento J V, et al. Developmental Assets in Adolescent Basketball Players: Influence of Age, Maturation, Size and Functional Capacities. Revista De Psicologia Del Deporte. 2019;28:46–52.

86. Krause LM, Naughton GA, Denny G, Patton D, Hartwig T, Gabbett TJ. Understanding mismatches in body size, speed and power among adolescent rugby union players. J Sci Med Sport. 2015;18(3):358–63.

87. Landgraff HW, Riiser A, Lihagen M, Skei M, Leirstein S, Hallén J. Longitudinal changes in maximal oxygen uptake in adolescent girls and boys with different training backgrounds. Scand J Med Sci Sports. 2021;31(S1):65–72.

88. Leão C, Silva AF, Badicu G, Clemente FM, Carvutto R, Greco G, et al. Body Composition Interactions with Physical Fitness: A Cross-Sectional Study in Youth Soccer Players. Int J Environ Res Public Health. 2022;19(6).

89. Lehnert M, Urban J, Procházka J, Psotta R. Isokinetic strength of knee flexors and extensors of adolescent soccer players and its changes based on movement speed and age. Acta Univ Palacki Olomuc, Gymn. 2011;41(2):45–33.

90. Leiter JR, Cordingley DM, MacDonald PB. Development of Anaerobic Fitness in Top-Level Competitive Youth Ice Hockey Players. J Strength Cond Res. 2018;32(9):2612–5.

91. Lemos LF, Oliveira VC, Duncan MJ, Ortega JP, Martins CM, Ramirez-Campillo R, et al. Physical fitness profile in elite beach handball players of different age categories. J Sports Med Phys Fitness. 20200630th ed. 2020;60(12):1536–43.

92. Leppänen M, Uotila A, Tokola K, Forsman-Lampinen H, Kujala UM, Parkkari J, et al. Players with high physical fitness are at greater risk of injury in youth football. Scand J Med Sci Sports. 2022;32(11):1625–38.

93. Letter RT, Dwyer DB, Drinkwater EJ, Feros SA. The Physical Qualities of Elite Australian Pace Bowlers: Typical Characteristics and Longitudinal Changes in Men and Women. Int J Sports Physiol Perform. 2022;17(12):1691–7.

94. Leyhr D, Kelava A, Raabe J, Höner O. Longitudinal motor performance development in early adolescence and its relationship to adult success: An 8-year prospective study of highly talented soccer players. PLoS One. 2018;13(5):e0196324.

95. Leyhr D, Raabe J, Schultz F, Kelava A, Höner O. The adolescent motor performance development of elite female soccer players: A study of prognostic relevance for future success in adulthood using multilevel modelling. J Sports Sci. 2020;38(11–12):1342–51.

96. Manuel Clemente F, Conte D, Sanches R, Moleiro CF, Gomes M, Lima R. Anthropometry and fitness profile, and their relationships with technical performance and perceived effort during small-sided basketball games. Res Sports Med. 2019;27(4):452–66.

97. Lolli L, Johnson A, Monaco M, Di Salvo V, Gregson W. Relative Skeletal Maturity and Performance Test Outcomes in Elite Youth Middle Eastern Soccer Players. Med Sci Sports Exerc. 2022;54(8):1326–34.

98. Loturco I, Jeffreys I, Abad CCC, Kobal R, Zanetti V, Pereira LA, et al. Change-of-direction, speed and jump performance in soccer players: a comparison across different age-categories. J Sports Sci. 2020;38(11–12):1279–85.

99. Łuszczyk M, Laskowski R, Ziemann E, Grzywacz T, Szczęsna-Kaczmarek A. Anaerobic Power and Dependence on Chosen Anthropometric Parameters in Young Handball Players. Balt J Health Phys Act. 2009;1(1).

100. Markovic G, Mikulic P. Discriminative ability of the Yo-Yo intermittent recovery test (level 1) in prospective young soccer players. J Strength Cond Res. 2011;25(10):2931–4.

101. Markovic G, Sarabon N, Boban F, Zoric I, Jelcic M, Sos K, et al. Nordic Hamstring Strength of Highly Trained Youth Football Players and Its Relation to Sprint Performance. J Strength Cond Res. 2020;34(3):800–7.

102. Martins PC, Teixeira AS, Antonacci Guglielmo LG, Francisco JS, Silva DAS, Nakamura FY, et al. Phase angle is related to 10 m and 30 m sprint time and repeated-sprint ability in young male soccer players. Int J Environ Res Public Health. 2021;18(9):4405.

103. Matthys SPJ, Vaeyens R, Fransen J, Deprez D, Pion J, Vandendriessche J, et al. A longitudinal study of multidimensional performance characteristics related to physical capacities in youth handball. J Sports Sci. 2013;31(3):325–34.

104. Matthys SPJ, Fransen J, Vaeyens R, Lenoir M, Philippaerts R. Differences in biological maturation, anthropometry and physical performance between playing positions in youth team handball. J Sports Sci. 2013;31(12):1344–52.

105. Mendez-Villanueva A, Buchheit M, Kuitunen S, Poon TK, Simpson B, Peltola E. Is the relationship between sprinting and maximal aerobic speeds in young soccer players affected by maturation? Pediatr Exerc Sci. 2010;22(4):497–510.

106. Mendez-Villanueva A, Buchheit M, Kuitunen S, Douglas A, Peltola E, Bourdon P. Age-related differences in acceleration, maximum running speed, and repeated-sprint performance in young soccer players. J Sports Sci. 2011;29(5):477–84.

107. Metaxas TI, Mandroukas A, Vamvakoudis E, Kotoglou K, Ekblom B, Mandroukas K. Muscle fiber characteristics, satellite cells and soccer performance in young athletes. J Sports Sci Med. 20140901st ed. 2014;13(3):493–501.

108. Metaxas T, Mandroukas A, Michailidis Y, Koutlianos N, Christoulas K, Ekblom B. Correlation of Fiber-Type Composition and Sprint Performance in Youth Soccer Players. J Strength Cond Res. 2019;33(10):2629–34.

109. Mirkov DM, Kukolj M, Ugarkovic D, Koprivica VJ, Jaric S. Development of anthropometric and physical performance profiles of young elite male soccer players: a longitudinal study. J Strength Cond Res. 2010;24(10):2677–82.

110. Morris R, Emmonds S, Jones B, Myers TD, Clarke ND, Lake J, et al. Seasonal changes in physical qualities of elite youth soccer players according to maturity status: comparisons with aged matched controls. Sci Med Football. 2018;2(4):272–80.

111. Nedeljkovic A, Mirkov DM, Kukolj M, Ugarkovic D, Jaric S. Effect of maturation on the relationship between physical performance and body size. J Strength Cond Res. 2007;21(1):245–50.

112. Nguyen AD, Zuk EF, Baellow AL, Pfile KR, DiStefano LJ, Boling MC. Longitudinal changes in hip strength and range of motion in female youth soccer players: Implications for ACL injury, A pilot study. J Sport Rehabil. 2017;26(5):358–64.

113. Niederer D, Damm M, Grigereit A, Banzer W, Vogt L. Game-specific abilities in elite youth football players: Validity and sensitivity to change of subjective coach ratings compared to objectively-assessed data. J Sports Med Phys Fitness. 2020;60(2):229–35.

114. Nikolaïdis PT. Cardiorespiratory power across adolescence in male soccer players. Hum Physiol. 2011;37(5):636–41.

115. Nikolaidis PT, Torres-Luque G, Chtourou H, Clemente-Suarez VJ, Ramírez-Vélez R, Heller J. Comparison between jumping vs. cycling tests of short-term power in elite male handball players: The effect of age. Movement and Sports Sciences - Science et Motricite. 2016;29(91):93–101.

116. Noon MR, Eyre ELJ, Ellis M, Myers TD, Morris RO, Mundy PD, et al. The influence of recruitment age and anthropometric and physical characteristics on the development pathway of English academy football players. Int J Sports Physiol Perform. 2021;16(2):199–207.

117. Nutt S, McKay MJ, Gillies L, Peek K. Neck strength and concussion prevalence in football and rugby athletes. J Sci Med Sport. 2022;25(8):632–8.

118. Nutton RW, Hamilton DF, Hutchison JD, Mitchell MJ, Simpson AHRW, MacLean JGB. Variation in physical development in schoolboy rugby players: Can maturity testing reduce mismatch? BMJ Open. 2012;2(4):e001149.

119. Parpa K, Michaelides M. Age-Related Differences in the Anthropometric and Physical Fitness Characteristics of Young Soccer Players: A Cross-Sectional Study. Children. 2022;9(5):650.

120. Peek K, Gatherer D, Bennett KJM, Fransen J, Watsford M. Muscle strength characteristics of the hamstrings and quadriceps in players from a high-level youth football (soccer) Academy. Res Sports Med. 2018;26(3):276–88.

121. Perroni F, Pintus A, Frandino M, Guidetti L, Baldari C. Relationship Among Repeated Sprint Ability, Chronological Age, and Puberty in Young Soccer Players. J Strength Cond Res. 2018;32(2):364–71.

122. Petridis L, Utczás K, Tróznai Z, Kalabiska I, Pálinkás G, Szabó T. Vertical Jump Performance in Hungarian Male Elite Junior Soccer Players. Res Q Exerc Sport. 2019;90(2):251–7.

123. Pizzigalli L, Cremasco MM, Torre A La, Rainoldi A, Benis R. Hand grip strength and anthropometric characteristics in Italian female national basketball teams. J Sports Med Phys Fitness. 2017;57(5):521–8.

124. Quatman CE, Ford KR, Myer GD, Hewett TE. Maturation leads to gender differences in landing force and vertical jump performance: A longitudinal study. Am J Sports Med. 2006;34(5):806–13.

125. Read PJ, Oliver JL, De Ste Croix MBA, Myer GD, Lloyd RS. Hopping and Landing Performance in Male Youth Soccer Players: Effects of Age and Maturation. Int J Sports Med. 20170920th ed. 2017;38(12):902–8.

126. Read PJ, Oliver JL, Myer GD, De Ste Croix MBA, Belshaw A, Lloyd RS. Altered landing mechanics are shown by male youth soccer players at different stages of maturation. Phys Ther Sport. 2018;33:48–53.

127. Rebelo-Gonçalves R, Coelho-e-Silva MJ, Valente-dos-Santos J, Tessitore A, Figueiredo AJ. Longitudinal study of aerobic performance and soccer-specific skills in male goalkeepers aged 11–18 years. Sci Med Football. 2017;1(1):40–7.

128. Reinikainen T, Burkett B, McKean M. Descriptive seasonal performance characteristics of junior elite Australian rules footballers aged twelve to seventeen. J Austral Strength Cond. 2015;23(4):12–9.

129. Ritsche P, Bernhard T, Roth R, Lichtenstein E, Keller M, Zingg S, et al. M. biceps femoris architecture and sprint ability in youth soccer players: a cross-sectional analysis. Int J Sports Physiol Perform. 2021;16(11):1616–24.

130. Roe M, Malone S. Yo-Yo Intermittent Recovery Test Performance in Subelite Gaelic Football Players From Under Thirteen to Senior Age Groups. J Strength Cond Res. 2016;30(11):3187–93.

131. Roe M, Malone S, Delahunt E, Collins K, Gissane C, Persson UM, et al. Eccentric knee flexor strength profiles of 341 elite male academy and senior Gaelic football players: Do body mass and previous hamstring injury impact performance? Phys Ther Sport. 20180302nd ed. 2018;31:68–74.

132. Roescher CR, Elferink-Gemser MT, Huijgen BCH, Visscher C. Soccer endurance development in professionals. Int J Sports Med. 2010;31(3):174–9.

133. Salinero JJ, Gonzalez-Millan C, Gutierrez D, Abian-Vicen J, Burillo P, Del Coso J. Age-related trends in anthropometry and jump and sprint performances in elite soccer players from 13 to 20 years of age: A cross-sectional study. Journal of Human Sport and Exercise. 2019;14(4):772–83.

134. Sander A, Keiner M, Wirth K, Schmidtbleicher D. Influence of a 2-year strength training programme on power performance in elite youth soccer players. Eur J Sport Sci. 2013 Sep;13(5):445–51.

135. Saward C, Hulse M, Morris JG, Goto H, Sunderland C, Nevill ME. Longitudinal Physical Development of Future Professional Male Soccer Players: Implications for Talent Identification and Development? Front Sports Act Living. 2020;2.

136. Sekine Y, Hoshikawa S, Hirose N. Longitudinal age-related morphological and physiological changes in adolescent male basketball players. J Sports Sci Med. 2019;18:751–7.

137. Silva AF, Alvurdu S, Akyildiz Z, Clemente FM. Relationships of Final Velocity at 30-15 Intermittent Fitness Test and Anaerobic Speed Reserve with Body Composition, Sprinting, Change-of-Direction and Vertical Jumping Performances: A Cross-Sectional Study in Youth Soccer Players. Biology (Basel). 2022;11(2):197.

138. Śliwowski R, Andrzejewski M, Wieczorek A, Barinow-Wojewódzki A, Jadczak, Adrian J, et al. Changes in the anaerobic threshold in an annual cycle of sport training of young soccer players. Biol Sport. 2013;30(2):137–43.

139. Söderman K, Bergström E, Lorentzon R, Alfredson H. Bone mass and muscle strength in young female soccer players. Calcif Tissue Int. 2000;67(4):297–303.

140. Spencer M, Pyne D, Santisteban J, Mujika I. Fitness Determinants of Repeated-Sprint Ability in Highly Trained Youth Football Players. Int J Sports Physiol Perform. 2011;6(4):497–508.

141. Stojmenović T, Ćurčić D, Vukašinović-Vesić M, Andjelković M, Dikić N, Kostić-Vučićević M, et al. Changes in maximal oxygen uptake during growth and development in girls who actively participate in basketball and non-athletes girls: A longitudinal study. Vojnosanit Pregl. 2018;75(5):481–6.

142. Sugimoto D, Borg DR, Brilliant AN, Meehan WP, Micheli LJ, Geminiani ET. Effect of sports and growth on hamstrings and quadriceps development in young female athletes: Cross-sectional study. Sports. 2019;7(7):158.

143. Saavedra JM, Kristjánsdóttir H, Einarsson IÞ, Guðmundsdóttir ML, Þorgeirsson S, Stefansson A. Anthropometric Characteristics, Physical Fitness, and Throwing Velocity in Elite women’s Handball Teams. J Strength Cond Res. 2018;32(8):2294–301.

144. Teixeira AS, Valente-Dos-Santos J, Coelho-E-Silva MJ, Malina RM, Fernandes-Da-Silva J, Cesar Do Nascimento Salvador P, et al. Skeletal Maturation and Aerobic Performance in Young Soccer Players from Professional Academies. Int J Sports Med. 2015;36(13):1069–75.

145. Te Wierike SCM, De Jong MC, Tromp EJY, Vuijk PJ, Lemmink KAPM, Malina RM, et al. Development of repeated sprint ability in talented youth basketball players. J Strength Cond Res. 2014;28(4):928–34.

146. Till K, Cobley S, O’Hara J, Chapman C, Cooke C. A longitudinal evaluation of anthropometric and fitness characteristics in junior rugby league players considering playing position and selection level. J Sci Med Sport. 2013;16(5):438–43.

147. Till K, Cobley S, Oʼhara J, Chapman C, Cooke C. An individualized longitudinal approach to monitoring the dynamics of growth and fitness development in adolescent athletes. J Strength Cond Res. 2013;27(5):1313–21.

148. Till K, Cobley S, O’ Hara J, Cooke C, Chapman C. Considering maturation status and relative age in the longitudinal evaluation of junior rugby league players. Scand J Med Sci Sports. 2014;24(3):569–76.

149. Till K, Tester E, Jones B, Emmonds S, Fahey J, Cooke C. Anthropometric and physical characteristics of english academy rugby league players. J Strength Cond Res. 2014;28(2):319–27.

150. Till K, Jones B, Emmonds S, Tester E, Fahey J, Cooke C. Seasonal changes in anthropometric and physical characteristics within English academy rugby league players. J Strength Cond Res. 2014;28(9):2689–96.

151. Till K, Jones B, Geeson-Brown T. Do physical qualities influence the attainment of professional status within elite 16-19 year old rugby league players? J Sci Med Sport. 2016;19(7):585–9.

152. Till K, Jones B, Darrall-Jones J, Emmonds S, Cooke C. Longitudinal development of anthropometric and physical characteristics within academy rugby league players. J Strength Cond Res. 2015;29(6):1713–22.

153. Till K, Jones B. Monitoring anthropometry and fitness using maturity groups within youth rugby league. J Strength Cond Res. 2015;29(3):730–6.

154. Till K, Cobley S, Morley D, O’Hara J, Chapman C, Cooke C. The influence of age, playing position, anthropometry and fitness on career attainment outcomes in rugby league. J Sports Sci. 20151029th ed. 2016;34(13):1240–5.

155. Till K, Morley D, O’Hara J, Jones BL, Chapman C, Beggs CB, et al. A retrospective longitudinal analysis of anthropometric and physical qualities that associate with adult career attainment in junior rugby league players. J Sci Med Sport. 2017;20(11):1029–33.

156. Toong T, Wilson KE, Urban K, Paniccia M, Hunt AW, Keightley M, et al. Grip Strength in Youth Ice Hockey Players: Normative Values and Predictors of Performance. J Strength Cond Res. 2018;32(12):3494–502.

157. Tribolet R, Bennett KJM, Watsford ML, Fransen J. A multidimensional approach to talent identification and selection in high-level youth Australian Football players. J Sports Sci. 20180426th ed. 2018;36(22):2537–43.

158. Vaeyens R, Malina RM, Janssens M, Van Renterghem B, Bourgois J, Vrijens J, et al. A multidisciplinary selection model for youth soccer: the Ghent Youth Soccer Project. Br J Sports Med. 20060915th ed. 2006;40(11):928–34; discussion 934.

159. Valente-Dos-Santos J, Coelho-E-Silva MJ, Martins RA, Figueiredo AJ, Cyrino ES, Sherar LB, et al. Modelling developmental changes in repeated-sprint ability by chronological and skeletal ages in young soccer players. Int J Sports Med. 2012;33(10):773–80.

160. Valente-dos-Santos J, Coelho-E-Silva MJ, Vaz J, Figueiredo F, Capranica L, Sherar LB, et al. Maturity-associated variation in change of direction  and dribbling speed in early pubertal years and 5-year  developmental changes in young soccer players. J Sports Med Phys Fitness. 2014;54(3):307–16.

161. Valente-dos-Santos J, Coelho-e-Silva MJ, Duarte J, Figueiredo AJ, liparotti JR, sherar LB, et al. Longitudinal predictors of aerobic performance in adolescent soccer players. Medicina (Kaunas). 2012;48(8):410–6.

162. Valente-dos-Santos J, Coelho-e-Silva M, Duarte J, Pereira J, Rebelo-Gonçalves R, Figueiredo A, et al. Allometric multilevel modelling of agility and dribbling speed by skeletal age and playing position in youth soccer players. Int J Sports Med. 20140415th ed. 2014;35(9):762–71.

163. Vandendriessche JB, Vaeyens R, Vandorpe B, Lenoir M, Lefevre J, Philippaerts RM. Biological maturation, morphology, fitness, and motor coordination as part of a selection strategy in the search for international youth soccer players (age 15-16 years). J Sports Sci. 2012;30(15):1695–703.

164. Vargas VZ, Motta C, Peres B, Vancini RL, Andre Barbosa De Lira C, Andrade MS. Knee isokinetic muscle strength and balance ratio in female soccer players of different age groups: a cross-sectional study. Phys Sportsmed. 20190802nd ed. 2020;48(1):105–9.

165. Vera-Assaoka T, Ramirez-Campillo R, Alvarez C, Garcia-Pinillos F, Moran J, Gentil P, et al. Effects of Maturation on Physical Fitness Adaptations to Plyometric Drop Jump Training in Male Youth Soccer Players. J Strength Cond Res. 2020;34(10):2760–8.

166. Vernillo G, Silvestri A, La Torre A. The yo-yo intermittent recovery test in junior basketball players according to performance level and age group. J Strength Cond Res. 2012;26(9):2490–4.

167. Visnapuu M, Jürimäe T. Relations of anthropometric parameters with scores on basic and specific motor tasks in young handball players. Percept Mot Skills. 2009;108(3):670–6.

168. Waldron M, Worsfold PR, Twist C, Lamb K. The relationship between physical abilities, ball-carrying and tackling among elite youth rugby league players. J Sports Sci. 2014;32(6):542–9.

169. Waldron M, Worsfold P, Twist C, Lamb K. Changes in anthropometry and performance, and their interrelationships, across three seasons in elite youth rugby league players. J Strength Cond Res. 2014;28(11):3128–36.

170. Williams CA, Oliver JL, Faulkner J. Seasonal monitoring of sprint and jump performance in a soccer youth academy. Int J Sports Physiol Perform. 2011;6(2):264–75.

171. Wollin M, Thorborg K, Welvaert M, Pizzari T. In-season monitoring of hip and groin strength, health and function in elite youth soccer: Implementing an early detection and management strategy over two consecutive seasons. J Sci Med Sport. 2018;21(10):988–93.

172. Bennett N, Woodcock S, Pluss MA, Bennett KJM, Deprez D, Vaeyens R, et al. Forecasting the development of explosive leg power in youth soccer players. Sci Med Football. 2019;3(2):131–7.

173. Woods CT, Keller BS, McKeown I, Robertson S. A Comparison of Athletic Movement Among Talent-Identified Juniors From Different Football Codes in Australia: Implications for Talent Development. J Strength Cond Res. 2016;30(9):2440–5.

174. Wright MD, Atkinson G. Changes in Sprint-Related Outcomes During a Period of Systematic Training in a Girls’ Soccer Academy. J Strength Cond Res. 2019;33(3):793–800.

175. Yang S, Chen H. Physical characteristics of elite youth male football players aged 13-15 are based upon biological maturity. PeerJ. 2022;10:e13282.
