# Supplementary material for: The development of physical characteristics in adolescent team sport athletes: A systematic review
Source: PLoS One. 2023 Dec 21;18(12):e0296181. doi: 10.1371/journal.pone.0296181 (PMC10735042; doi:10.1371/journal.pone.0296181)
Supplement: S4 Table — CMJ: countermovement jump. CoD: change of direction. (DOCX) [file pone.0296181.s005.docx]

|  |  | **U12** | **U13** | **U14** | **U15** | **U16** | **U17** | **U18** | **U19** | **U20** |
| --- | --- | --- | --- | --- | --- | --- | --- | --- | --- | --- |
| **Boys** |  |  |  |  |  |  |  |  |  |  |
|  | **10 m (n)** | 10 | 30 | 46 | 52 | 34 | 23 | 10 | 12 | 4 |
|  | **30 m (n)** | 13 | 27 | 42 | 51 | 31 | 16 | 4 | 5 | 1 |
|  | **CoD (n)** | 7 | 14 | 32 | 35 | 25 | 11 | 5 | 2 |  |
|  | **CMJ (n)** | 33 | 27 | 50 | 47 | 61 | 35 | 27 | 16 | 6 |
|  | **Intermittent endurance (n)** | 29 | 30 | 39 | 35 | 31 | 25 | 19 | 9 | 2 |
|  | **Weight (n)** | 78 | 88 | 128 | 127 | 117 | 79 | 51 | 33 | 9 |
|  | **Height (n)** | 78 | 84 | 127 | 124 | 116 | 75 | 49 | 29 | 7 |
|  | **Body fat (n)** | 20 | 30 | 42 | 41 | 41 | 27 | 19 | 8 | 2 |
|  | **Handgrip (n)** | 7 | 11 | 11 | 15 | 8 | 6 | 4 | 2 | 2 |
|  | **Lower body strength (n)** | 12 | 11 | 11 | 9 | 11 | 13 | 6 | 6 | 3 |
| **Girls** |  |  |  |  |  |  |  |  |  |  |
|  | **10 m (n)** | 3 | 2 | 2 | 4 | 2 |  | 2 | 1 |  |
|  | **30 m (n)** | 3 | 3 | 1 | 3 | 1 |  | 1 |  |  |
|  | **CoD (n)** | 1 | 1 | 1 | 2 | 1 |  |  |  |  |
|  | **CMJ (n)** | 2 | 3 | 2 | 5 | 2 |  | 1 |  |  |
|  | **Intermittent endurance (n)** | 4 | 4 | 2 | 3 | 1 |  |  |  |  |
|  | **Weight (n)** | 10 | 10 | 16 | 15 | 13 | 5 | 5 | 2 | 1 |
|  | **Height (n)** | 10 | 10 | 16 | 15 | 13 | 5 | 5 | 2 | 1 |
|  | **Body fat (n)** | 2 | 1 | 4 | 5 | 3 | 1 | 1 | 1 |  |
|  | **Handgrip (n)** | 3 | 3 | 3 | 3 | 1 |  |  |  | 1 |
|  | **Lower body strength (n)** | 2 | 1 | 3 | 2 | 3 | 1 | 1 |  |  |
