# Supplementary figures and images for: The development of physical characteristics in adolescent team sport athletes: A systematic review
Source: PLoS One. 2023 Dec 21;18(12):e0296181. doi: 10.1371/journal.pone.0296181 (PMC10735042; doi:10.1371/journal.pone.0296181)

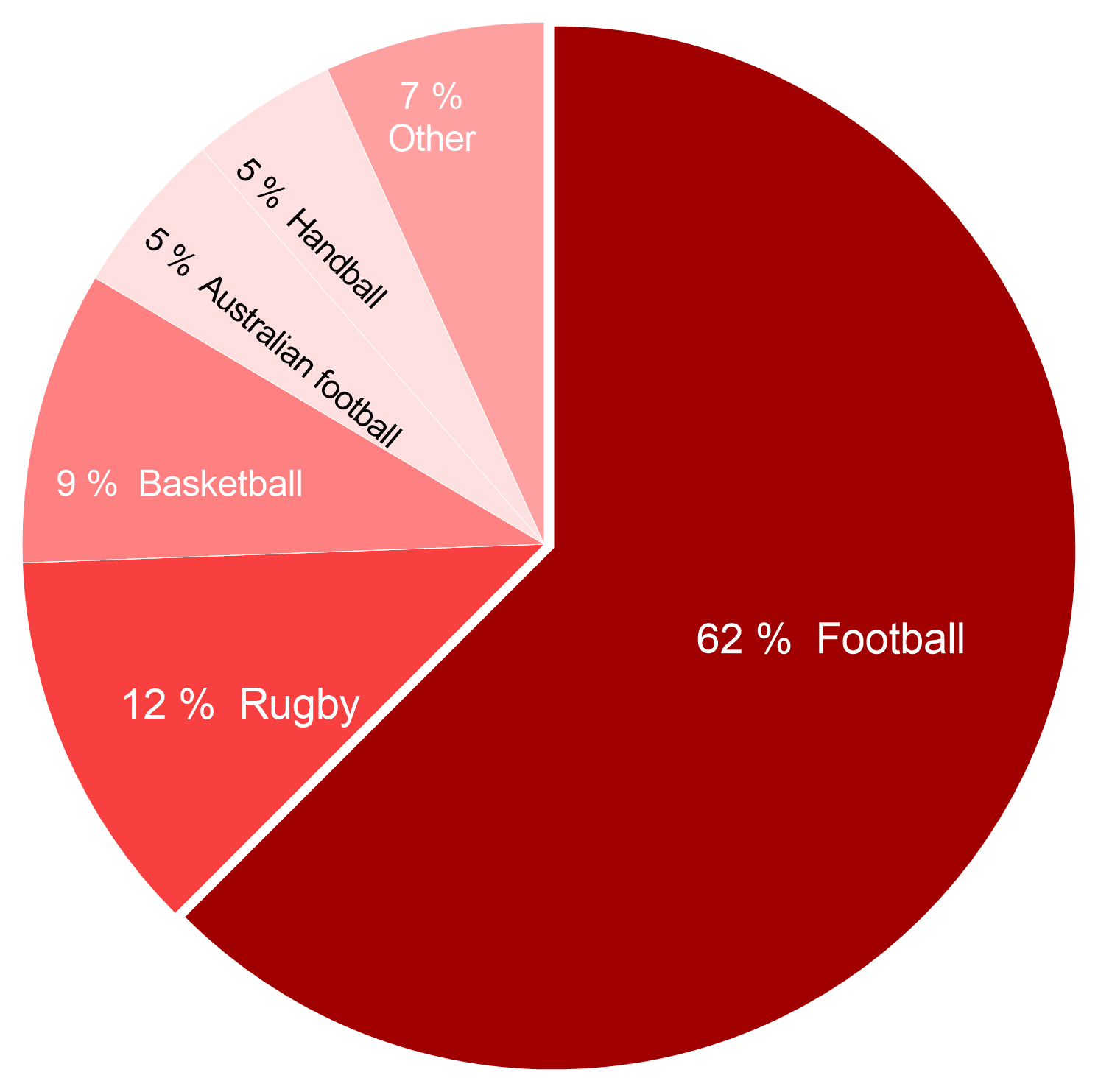

Supplement: S1 Fig — (TIF) [file pone.0296181.s007.tif]
